# On the capacity for rapid adaptation and plastic responses to herbivory and intraspecific competition in insular populations of *Plectritis congesta*

**DOI:** 10.1111/eva.13371

**Published:** 2022-04-08

**Authors:** Cora L. Skaien, Peter Arcese

**Affiliations:** ^1^ Department of Forest and Conservation Sciences Faculty of Forestry University of British Columbia Vancouver British Columbia Canada

**Keywords:** evolvability, genetic variance, local adaptation, migration‐selection balance, phenotypic plasticity, ungulate herbivory

## Abstract

A capacity for rapid adaptation should enhance the persistence of populations subject to temporal and spatial heterogeneity in natural selection, but examples from nature remain scarce. *Plectritis congesta* (*Caprifoliaceae*) is a winter annual that exhibits local adaptation to browsing by ungulates and hypothesized to show context‐dependent trade‐offs in traits affecting success in competition versus resistance or tolerance to browsing. We grew P. congesta from 44 insular populations historically exposed or naïve to ungulates in common gardens to (1) quantify genetic, plastic and competitive effects on phenotype; (2) estimate a capacity for rapid adaptation (evolvability); and (3) test whether traits favoured by selection with ungulates present were selected against in their absence. Plants from browsed populations bolted and flowered later, had smaller inflorescences, were less fecund and half as tall as plants from naïve populations on average, replicating patterns in nature. Estimated evolvabilities (3–36%) and narrow‐sense heritabilities (h2; 0.13–0.32) imply that differences in trait values as large as reported here can arise in 2–18 generations in an average population. Phenotypic plasticity was substantial, varied by browsing history and fruit phenotype and increased with competition. Fecundity increased with plasticity in flowering height given competition (0.47 ± 0.02 florets/cm, β ± se), but 23–77% faster in naïve plants bearing winged fruits (0.53 ± 0.04) than exposed‐wingless plants (0.43 ± 0.03) or exposed‐winged and naïve‐wingless plants (0.30 ± 0.03, each case). Our results support the hypothesis that context‐dependent variation in natural selection in *P*. *congesta* populations has conferred a substantial capacity for adaptation in response to selection in traits affecting success in competition versus resistance or tolerance to browsing in the absence versus presence of ungulates, respectively. Theory suggests that conserving adaptive capacity in *P*. *congesta* will require land managers to maintain spatial heterogeneity in natural selection, prevent local extinctions and maintain gene flow.

## 1 INTRODUCTION

Understanding how spatial and temporal variation in natural selection influence quantitative and plastic traits affecting fitness are shared goals in conservation and evolutionary biology and essential to predicting how locally adapted populations may respond to environmental change in future (e.g. Bonnet et al., 2022; Chevin et al., 2010; Cronk, 2016; DeMarche et al., 2019; Fox et al., 2019; Franks et al., 2014; Hendry et al., 2018; Ho & Zhang, 2018; Lande & Shannon, 1996). However, applying such predictions can imply substantial knowledge of the biotic and abiotic factors that may act as selective agents above and belowground, the direct and indirect responses to selection of phenotypic traits affecting fitness and the resulting capacity of populations to persist in situ given ongoing environmental change. Such uncertainties make detailed empirical studies of adaptive evolution valuable to conservation planning (e.g. Bonnet et al., 2022; Franks et al., 2014; Hendry et al., 2018; Olivieri et al., 2016; Radichuck et al., 2019; Santangelo et al., 2020), particularly for species on islands (Cronk, 2016; Grant et al., 2017; Stuart et al., 2014), which comprise 6.8% of land area but host most of the world's threatened species (Fernández‐Palaciosa, 2021; Gray, 2019; Spatz et al., 2017).

We focus here on the conservation of plant populations subject to variation in the presence or absence of browsing ungulates, which can decimate species richness and cover where predators have been eradicated or browsers colonize islands without them (e.g. Arcese et al., 2014; Caughley, 1970; Estes et al., 2011; Martin et al., 2010). Despite much evidence of local adaptation in plant populations in response to herbivory (e.g. Ågren et al., 2013; Carey, 1983; Lennartsson et al., 1997; Paige & Whitham, 1987), the potential pace of phenotypic change, its dependence on environmental context and underlying mechanisms often remain cryptic (Didiano et al., 2014; López‐Goldar & Agrawal, 2021; Shaw, 2019; Turley et al., 2013). Moreover, because phenotypic plasticity and adaptive evolution can each influence persistence via the expression of context‐dependent traits (Hendry, 2015), but only adaptive evolution can ensure the persistence of populations subject to ongoing change (e.g. Bonnet et al., 2022; Duputié et al., 2015; Fox et al., 2019; van Kleunen & Fischer, 2005), quantifying both should advance conservation practice. For example, restoring gene flow to locally adapted populations experiencing genetic deterioration is predicted to enhance population mean fitness and lower extinction risk by increasing additive genetic variance and facilitating adaptive evolution in highly variable, auto‐correlated environments (*cf* Lande & Shannon, 1996; see also Franks et al., 2014; Cronk, 2016; Bell, 2017; Shaw, 2019). However, doing so in unpredictable environments or populations displaying ‘perfect local adaptation’ (Palacio‐López et al., 2015) may reduce these desirable conservation outcomes by admixing populations adapted to opposite selective environments (e.g. Bell, 2017; Emery, 2009; Lande & Shannon, 1996; Shaw, 2019), emphasizing the value of empirical knowledge.

Context‐dependent trade‐offs in the fitness value of traits affecting resistance or tolerance to herbivory, growth rate and competitive ability are also widely reported in plant populations (e.g. Agrawal, 2011; López‐Goldar & Agrawal, 2021; Turley et al., 2013; Züst & Agrawal, 2017). For example, Johnson et al. (2009) described trade‐offs in the fitness value of quantitative and plastic traits linked to growth in response to herbivory by insects in evening primrose (*Oenothera biennis*), and also shown to vary by plant genotype in the presence or absence of deer (*Cervidae*) and voles (*Cricetidae*; Parker et al., 2010). Deer also drove context dependence in the fitness value of branching versus upright growth in Scarlet gilia (*Impomopsis aggregata*, Paige & Whitham, 1987; Juenger & Bergelson, 2000), and divergence in insular populations of western redcedar (*Thuja plicata*) in chemical defence and branching habit (Stroh et al., 2008; Vourc’h et al., 2001), *Primula farinose* in stipe length (Ågren et al., 2013) and short‐spurred seablush (*Plectritis congesta*) in growth habit, height, flowering phenology, inflorescence size and fruit phenotype (Skaien & Arcese, 2018, 2020). However, because herbivory is one of many disturbance agents potentially affecting plant phenotype and environmental context, empirical studies of the genetic and plastic components of variation in traits affecting resistance or tolerance of herbivory can help to elucidate mechanisms (e.g. Archibald et al., 2019; López‐Goldar & Agrawal, 2021).

Here, we consider further how insular populations of *P*. *congesta* adapted or naïve to browsing ungulates might respond to variation in browsing pressure by: (1) estimating additive genetic (V_a_) and phenotypic (V_p_) variances, narrow‐sense heritabilites and capacity for rapid adaptation (i.e. ‘evolvability’; Houle, 1992) in island populations of *P*. *congesta* historically exposed or naïve to browsing ungulates; (2) test whether traits favoured by natural selection in the presence of browsers ware selected against in their absence; and (3) test for context‐dependent trade‐offs in plant height and fecundity thought to arise in nature due to variation in plant density in the presence or absence of browsers (Skaien & Arcese, 2020). *P*. *congesta* is an iconic winter annual of threatened oak–savanna habitat in western North America, a key resource for native pollinators (e.g. Adderley & Vamosi, 2015; Kelly & Elle, 2020; Schultz et al., 2010), and ideal for our purposes. *P*. *congesta* displays marked local adaptation to browsing and context‐dependent trade‐offs in the fitness value of traits shown to drive population growth and persistence in the presence or absence of deer (Skaien & Arcese, 2020), a pattern thought to occur in other plant‐herbivore systems (e.g. López‐Goldar & Agrawal, 2021; McGoey & Stinchcombe, 2009; Ramos & Schiestl, 2020; Wenk & Falster, 2015).

Briefly, surveys of 285 island and mainland populations and field estimates of population growth and persistence in 13 translocated populations implicate browsing by ungulates as a key factor affecting local adaptation in plant phenology, morphology, genotype and fitness in *P*. *congesta* (Gonzales & Arcese, 2008; Skaien & Arcese, 2020). Notably, each *P*. *congesta* plant produces one of two fruit phenotypes, with or without wing‐like appendages (Jacobs et al., 2010), following Mendelian dominance at one locus (Ww, WW = winged, ww = wingless, Ganders et al., 1977). Plants in situ and grown from islands with resident ungulates grew as rosettes in early life, germinated and bolted later, produced mainly wingless fruits and small terminal inflorescences; opposite to patterns observed in plants *in situ* and from islands without ungulates (Skaien & Arcese, 2018, 2020). Moreover, Carey (1983) used artificial selection to demonstrate rapid adaptation in plant height in *P*. *congesta* from two populations to increase or reduce plant height 150 or 50% in five generations, respectively. *P*. *congesta* also exhibited plasticity in height, branching and flower morphology when grown in environmental chambers (Carey & Ganders, 1980), and latitudinal variation in flowering phenology in nature (Reed et al., 2019). However, V_a_, V_p_, narrow‐sense heritability (h^2^), and the capacity of *P*. *congesta* populations to evolve in response to temporal and spatial variation in browsing remain unknown.

In common gardens absent of browsers, we expected that traits shown previously to enhance population persistence in the presence of deer (e.g. delayed germination, bolting, and flowering and the production of wingless fruits on small terminal inflorescences) might represent a disadvantage to plants adapted to browsing, particularly given increasing competition with neighbours naïve to it. We also expected plants from populations historically exposed to browsers to germinate, bolt and flower later, and produce more total and basal branches than *P*. *congesta* from naïve populations, thus replicating our prior results from nature. We further expected such traits to display substantial additive genetic variance (V_a_), narrow‐sense heritabilities (h^2^) and high evolvabilities given much evidence that gene flow between populations facing opposite selection pressures drives spatial variation in fruit phenotype and genotype in the islands we study and reflect a history of temporal and spatial variation in ungulate occurrence (e.g. Arcese et al., 2014; Gonzales & Arcese, 2008; Skaien & Arcese, 2018, 2020).

By contrast, predicting outcomes with respect to fruit phenotype in common gardens sown at high density is more challenging given patterns observed in nature. For example, in split‐plot common gardens open to and protected from deer and sown at 0.75‐m spacing, plants bearing wingless fruits expressed much higher fitness than those bearing winged fruits in the presence of deer (Skaien & Arcese, 2020), as expected given their high relative abundance on islands with versus without browsers (73 vs. 9%, respectively; Skaien & Arcese, 2018). Specifically, when exposed to ungulate herbivores, plants bearing wingless fruits and historically exposed to browsing had 200–500% higher survival and had 180% higher fecundity than plants from naïve populations or those bearing winged fruits (Skaien & Arcese, 2020). By contrast, although fruit phenotype did not predict the survival or fecundity of individual plants in the absence of deer, plants bearing winged fruits and from historically exposed populations were 20% less fecund than naïve plants also bearing winged fruits on average (Skaien & Arcese, 2020). Over 5 years, the differences above contributed to plants bearing winged fruits and derived from naïve populations becoming 300 to 480% more abundant than other groups in the absence of deer. Meanwhile, plants naïve to browsing, bearing winged fruits and thus expressing the dominant W allele underlying the polymorphism were extirpated within 3 years when available to deer (Skaien & Arcese, 2020). Overall, therefore, we expected plants grown from wingless fruits and from populations historically exposed to deer to express lower fecundity, later germination, bolting and flowering, and smaller inflorescences mainly due to pleiotropy or linkage at the fruit wing locus (*cf* Carey & Ganders, 1980; Skaien & Arcese, 2020). However, because such traits may also represent a disadvantage to plants adapted to browsing in competition with naïve neighbours, we also expected to observe plasticity in flowering height as the number and height of adjacent neighbours increased, especially in plants historically exposed to browsing and grown from or expressing wingless fruits. Last, because we also expected that resource limitation might force trade‐offs between growth and fecundity in individual plants, and to be most evident in plants adapted to browsing and grown from or bearing wingless fruits, we expected fecundity to decline as plasticity in flowering height increased in response to the number, height and origin of adjacent competitors.

## 2 MATERIALS AND METHODS

### 2.1 Fruit collection

We collected *P*. *congesta* fruits, which each contain a single seed, from 44 populations (n = 16 historically exposed; n = 28 historically naïve) in southwestern British Columbia, Canada, in one or more years (Appendix S1; 20 in 2005, 17 in 2006, 21 in 2015). ‘Historically exposed’ populations host resident deer (*Dama* and/or *Odocoileus hemionus*, on all but one island) or sheep (*Ovis aries*, one island), whereas ‘historically naïve’ populations occur on islands with no sign of ungulates (e.g. pellets, browsed plants) in ≥20 years and/or with shoreline cliffs preventing easy access (*cf* Martin et al., 2015). *P*. *congesta* is self‐compatible, with average outcrossing rates of 48–80% across multiple populations (Ganders et al., 1977).

In 35 of 44 populations, we collected fruits from 20 plants within each population as field‐pollinated maternal sibships (hereafter, ‘families’, including all fruits collected from a single maternal plant) to allow estimates of genetic variance, heritability, evolvability, and additive genetic and phenotypic coefficients of variation (Appendix S1). In 2005, we also collected ‘pooled’ samples of fruits in 20 of 44 populations, comprising the fruits of 10–20 field‐pollinated plants spaced ≥1 m apart and selected to represent the range of variation in fruit hue, size, hairiness and wing phenotype observed within populations (Appendix S1), which differed visibly among individual plants. Pooled populations were included in analyses of plant phenology and phenotype to increase statistical power in relation to browsing history, ensure that all beds were fully occupied and to include replicate populations in gardens grown in different years. Because each plant produced fruits of a single phenotype and was originally selected to maximize variation in the traits noted, we felt confident that 7–10 maternal plants from each pooled sample employed were about equally represented across beds. All fruits were sun‐dried, then stored in paper envelopes at 5°C.

### 2.2 Experimental design

Two common gardens were planted (2006–07 and 2015–16, same raised beds) to replicate results, increase the range of environmental conditions in source populations, and increase sample sizes and precision. Eight populations were planted in both gardens to help account for annual variation. In 2006–07, we used fruits collected in 2005 and 2006 from 17 populations collected as families and 20 ‘pooled’ populations (7–12 families/population; Appendix S1). In 2015–16, we only sowed fruits collected as families in 2015 (21 populations and ~12 families/population; Appendix S1). In all cases, fruits were sown 10 cm apart in raised beds (1.2 x 2.4 m; 12 in 2006–07, 15 in 2015–16) filled with sandy loam, located at Totem Field, University of British Columbia, Canada. Fruits were planted in randomized blocks, with 7–12 families/bed/population, totalling 1442 winged and 625 wingless fruits in 2006–07, and 1713 winged and 1311 wingless fruits in 2015–16. We included 30–44 fruits per population from pooled samples in 2006–07. In both years, all beds were protected from ungulates and slugs and showed little evidence of damage by insects or vertebrates.

Planting occurred September 25 to October 5 (2006) and September 20 to 29 (2015). Beds were watered gently to secure fruits in soil upon planting, then every 3 days to 92 days when all plants had become established and a clear plastic canopy designed to prevent fruit displacement was removed. All beds were subject to natural variation in weather and weeded regularly thereafter.

Plant morphology and phenology were assessed the same days postplanting in 2006–07 and 2015–16 gardens, and timed to reflect planting order (Day 55, November; Day 119, January; Day 192, March). However, we advanced measurement in April and May of 2016 relative to 2007 to accommodate a warmer spring and more rapid maturation. Germination was assessed at day 55. Plant height and diameter (widest point) were recorded at days 55, 119 and 192 in all surviving plants, and height was recorded in April and May. We then used the height to width ratio (H:W ratio) to reflect an apparent continuum of rosette (H:W ratio <1) to tall growth forms. Flowering phenology was scored as buds absent or present, or when present as florets open or not (day 192). In May of 2007 and 2016, we counted all branches, the number <10 cm above ground, measured the height of the lowest branch, and recorded fruit phenotype. Because *P*. *congesta* can produce hundreds of florets per inflorescence and mature over days to weeks, estimating fecundity precisely was impractical. Instead, we recorded the height and width of terminal inflorescences in May 2015 (not 2006) to estimate the number of fruits based on inflorescence volume via regression (Appendix S1; Adjusted R^2^ = 0.50). Variation in the number of neighbours at each planting location varied naturally due to germination success and subsequent survival, and ranged from 0 neighbours (6 cases) to 8 neighbours.

### 2.3 Genetic variance, heritability, coefficients of variation and evolvability

We estimated the (1) additive genetic variance (V_a_; the additive genetic contribution to phenotype not accounting for dominance or epistasis); (2) narrow‐sense heritability (proportion of phenotypic variance explained by inheritance, h^2^; Visscher et al., 2008); (3) the coefficient of additive genetic variation (CV_A_; a measure of the additive genetic variation relative to the trait mean); (4) evolvability (the ability of a population to respond to natural selection, expressed as the predicted, percentage change in a trait per generation, depending on the strength of and response to selection; Hansen et al., 2003; Houle, 1992; Visscher et al., 2008); and (5) the coefficient of phenotypic variation (CV_p_; a measure of the relative phenotypic variation relative to the trait mean, including all genetic, environmental and residual variation). Only fruits collected in family units were used for these estimates, and both garden years were used.

We estimated the variance explained by the additive genetic component of genotype (V_a_) and phenotypic variance (V_p_) for (1) plant height (Day 192, natural log transformed), (2) growth form (H:W ratio; Day 192, natural log transformed) and (3) the number of branches below 10 cm (May) using linear mixed effects models and the lme4 package (Bates et al., 2015) in R (R 3.1.0 Statistic Package, R Core Team, 2014). Random effects included in all models were: (1) family nested within population of origin (V_f_); and (2) bed nested within garden year. Additive genetic variance (V_a_) was estimated from family variance (V_f_; obtained from the random effect variance for ‘family’ within the linear mixed effects models) under a mixed mating system, representing outcrossing rates reported for *P*. *congesta* by Ganders et al. (1977) of between 48 and 80%. Calculations assume that fruits from the same family are a mixture of half‐siblings (V_a_ = 4*V_f_) and full‐siblings (V_a_ = 2*V_f_), with the relative proportion of each represented at the extreme ends of the outcrossing rates previously reported (i.e. 0.52*2*V_f_ +0.48*4*V_f_, and 0.2*2*V_f_ +0.8*4*V_f_). We estimated narrow‐sense heritability as h^2^ = V_a_/V_P_ at both 48% and 80% outcrossing (Visscher et al., 2008). Phenotypic variance was estimated by combining variance explained from family and residual variance from the random effects variances in the linear mixed effects model. The coefficient of additive genetic variation was calculated as V_a_^0.5^/µ*_i_* and the coefficient of phenotypic variation was calculated as V_p_^0.5^/µ*_i_*, where µ*_i_* is the mean of trait *i* (Houle, 1992). We estimated evolvability as 100 * (V_a_ / (µ*_i_* ^2^)) at both 48% and 80% outcrossing (Houle, 1992). We then estimated the number of generations required for trait means in historically exposed populations to reach the trait mean in historically naïve populations in the Totem Field common garden, and vice versa, using the formula *N_gen_* = [((µ_1_ ‐ µ_2_) / µ_2_) * 100] / evolvability, where *N_gen_* is the number of generations, µ_1_ is the mean value observed for plants from historically exposed or naïve populations, and µ_2_ is the mean value observed in the opposite selective environment.

### 2.4 Trait variation relative to ungulate herbivory

We estimated the effects of: (1) browsing history (i.e. fruits originating from historically exposed or naïve populations); and (2) fruit phenotype of resultant plant (i.e. winged or wingless) on: (A) plant height at days 55, 119 and 192; (B) growth form (height to width ratio, H:W Ratio; greater values denote more allocation to upward than outward growth) at days 55, 119 and 192; (C) the number of total branches in May; (D) the number of branches below 10 cm in May; (E) height of the lowest branch in May; and (F) budding phenology at Day 192 (no buds, buds or blooming). These analyses were conducted using the glmmTMB package (Brooks et al., 2017) in R (R 3.1.0 Statistic Package, R Core Team, 2014) using all fruits planted in 2006–07 and 2015–16 (pooled and those in family units). Random effects included were population and bed ID nested within garden year. All model results are shown in appendices. All models were tested for goodness of fit and to ensure assumptions of the model were satisfied. Likelihood ratio tests were performed for each random effects variable, with results of all tests supporting the inclusion of population in the models (*p* < 0.0001 for all), and results from all tests but three suggesting that garden year was needed in the models (*p* < 0.0001 for most models; the three that did not require year were for plant height at day 192, H:W ratio at day 119, and the number of branches under 10 cm). When there was a significant interaction, we performed post hoc tests to differentiate between origin*fruit phenotype groups using the emmeans package (Lenth, 2022); post hoc tests were not required when comparing significant differences within a two‐factor variable.

We assessed plant height and growth form using separate analyses at each measurement date testing main effects of and two‐way interactions between browsing history and observed fruit phenotype (Appendix S1). Only plants that germinated and survived to each measurement date and were not damaged during sampling were used (December, n = 3408; all other dates, n = 3515). Models used Gaussian distributions and log transformations of height and the H:W ratio (representing growth form). Growth form was only estimated at days 55, 119 and 192. However, note that low H:W ratios observed later in the season (e.g. Day 192) may not indicate rosettes, given that some plants as tall as they are wide may have substantial volume via lateral branching. H:W ratio thus remained a useful metric of an individual plant's investment in vertical versus outward growth, a trait also thought to influence resistance and/or tolerance of ungulate herbivory in *P*. *congesta* (Skaien & Arcese, 2020).

We counted in May the total number of branches on plants and the number of branches <10 cm above ground level. We analysed data by including two‐way interactions between browsing history and observed fruit phenotype (Appendix S1), using a Gaussian distribution for total branches (natural log transformation), and Poisson distribution and log link for branches <10 cm. Although we acknowledge that using a Poisson or a negative binomial distribution is typically more appropriate for count data, the model for branch number in May using the log‐transformed data using a Gaussian distribution had the lowest AIC (even when accounting for corrections between distribution types) and performed best. Statistical comparison of lowest branch height was conducted using a Kruskal–Wallis nonparametric analysis of variance.

We assessed flowering phenology of surviving plants at Day 192 using the ‘multinom’ function in the nnet package (Venables & Ripley, 2002), using flowering phenology classes as multinomial responses (where no bud, bud present and blooming were 1–3, respectively) to test the two‐way interaction between browsing history and fruit phenotype (Appendix S1).

### 2.5 Intraspecific Competition, growth form, and plant height

We estimated phenotypic responses of height, H:W ratio, number of branches and fecundity to variation in number of adjacent neighbours in 2015–16 by taking advantage of natural variation in germination success, which varied about randomly across populations and beds, yielding an index from 0 to 8 (neighbours within 14.2 cm of focal plant). High germination success and lack of data on fecundity precluded use of the 2006–07 garden.

Fecundity was estimated as number of florets and/or fruits produced by each focal plant (derived from the regression equation; Appendix S1), and with the trait values above, evaluated by browsing history, fruit phenotype, number and the mean height of adjacent neighbours (with 0 neighbours assigned 0 cm height for neighbours). Specifically, in one model, we tested main effects and two‐way interactions between (1) browsing history and fruit phenotype of the focal plant, (2) the number of neighbours and proportion originating from historically exposed populations and (3) the number of neighbours and mean height of neighbouring plants (Appendix S1) on: (A) the number of fruits produced (negative binomial distribution; Appendix S1); (B) plant height in May (untransformed; Gaussian distribution; Appendix S1); (C) ‘growth form’ at day 192 (H:W ratio, natural log transformed; Gaussian distribution; Appendix S1); (D) total number of branches in May (Poisson distribution, log link; Appendix S1); and (E) the number of branches below 10 cm in May (Poisson distribution, log link; Appendix S1). Random effects included bed ID and family nested within population of origin in all models described here. All models were ran using the glmmTMB package (Brooks et al., 2017). We performed post hoc tests to differentiate between origin*fruit phenotype groups using the emmeans package (Lenth, 2022), using an alpha of 0.0125 (0.05 divided by 4 comparisons).

### 2.6 Plasticity, fecundity and flowering height

We quantified phenotypic variation in May flowering height in response to competitive environment (‘plasticity in height given competition’) as the difference between observed plant height in May minus its expected height in the absence of competition, expressed as a percentage (e.g. plasticity = ((ht_May_ – ht_exp_) / ht_exp_) * 100); *cf* Arnold et al., 2019; Valladares et al., 2006. Expected height was estimated by regressing May height on the number of adjacent competitors, fraction from naïve populations, and mean height in May using a linear mixed model (Gaussian distribution, untransformed fixed effects, bed as a random effect; Appendix S1). We then used a general linear mixed model (Gaussian distribution, untransformed fixed effects) to test our predictions with respect to fecundity, competition and plasticity in May height, given browsing history and fruit phenotype (see Introduction).

## 3 RESULTS

We scored 3544 plants for quantitative traits in two common gardens (n_2007,pooled_ = 480, n_2007,families_ = 1589, n_2015,families_ = 1507). Germination rates were high and similar among populations in 2006–07 (~76%), but lower in 2015–16 (~51%). Survival from germination to flowering was high in both gardens (95–98%). Partial residual plots of the influence of competition on modelled traits appear in Appendix S1.

### 3.1 Genetic variance, heritability, coefficients of variation and evolvability

Estimates of additive genetic variance and heritability were modest for growth form, intermediate for plant height and highest for the number of branches below 10 cm (Table 1). Coefficients of additive genetic variance were similar for all traits (CV_a_ = 17 to 60), with coefficients of phenotypic variance considerably larger (CV_p_ = 38 to 158; Table 1). Estimated of evolvabilities imply that change in traits values of the magnitude observed between historically browsed and naïve populations could occur in 2–18 generations (Table 1; Appendix S1).

**TABLE 1:** Mean trait values (natural log), additive genetic variance (V_a_), narrow‐sense heritability (h^2^), coefficient of variation for additive genetic variance (CV_a_) and phenotypic variance (CV_p_), evolvability, and the estimated number of generations to reach the mean value observed in populations exposed to opposite selective pressures with respect to browsing, for plant height, growth form (H:W Ratio) and the number of branches below 10 cm (see supplementary materials for details. Lower and upper bounds represent estimates across the spectrum of observed outcrossing (see Methods)

Trait	ln(*u_i_*) ± SD	V_a_	h^2^	CV_a_	CV_p_	Evolvability	#Generations
ln(Height D192)	7.4 ± 4.4	0.09–0.11	0.21–0.26	16.7– 18.4	38.5–39.3	2.79–3.39	10–18
ln(H:W Ratio D192)	0.8 ± 0.4	0.03–0.04	0.13–0.16	54.0–60.1	155.6–157.6	29.71–36.13	2–5
# Branches <10 cm	1.6 ± 0.8	0.20–0.25	0.26–0.32	27.7– 30.6	58.8–60.17	7.67 – 9.34	5–10

### 3.2 Trait variation and herbivory

**FIGURE 1:**
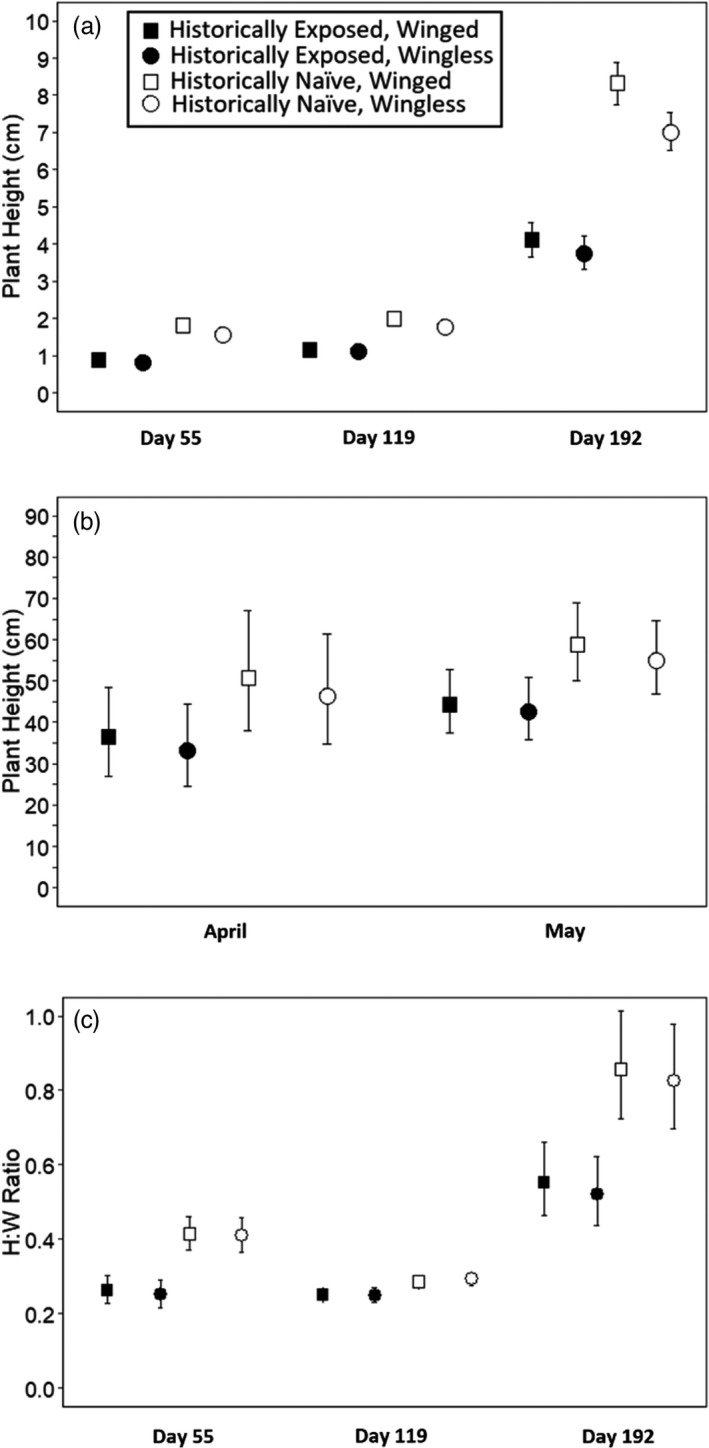
Plant height at 55, 119 and 192 days after planting (a), in April and May (b), and (c) growth form at days 55, 119, and 192 days after planting in both gardens combined (2006–07; 2015–16). Values represent back‐transformed means ± SEs, with differences between years accounted for with year as a random effect in models (glmmTMB; Appendix S1). Plants from historically naïve populations, and to a lesser degree those grown from winged fruits, tended to be taller than plants from historically exposed populations and those with wingless fruits (see inset)

Plants from historically naïve populations were ~28 to 40% taller than plants from exposed populations at all five censuses (Figure 1a,b; Appendix S1; Day 55: χ^2^ = 35.21, *p* < 0.0001; Day 119: χ^2^ = 61.08, *p* < 0.0001; Day 192: χ^2^ = 56.88, *p* < 0.0001, two‐way interaction browsing history and fruit phenotype, χ^2^ = 4.65, *p* = 0.03, post hoc tests all comparisons *p* < 0.0125; April: χ^2^ = 17.87, *p* < 0.0001; May: χ^2^ = 20.51, *p* < 0.0001). Plants bearing winged fruits were also 10 – 20% taller than plants bearing wingless fruits to Day 192, and 5–10% taller thereafter (Figure 1a,b; Appendix S1; Day 55: χ^2^ = 26.17, *p* < 0.0001; Day 119: χ^2^ = 24.72, *p* < 0.0001; Day 192: χ^2^ = 51.52, *p* < 0.0001, two‐way interaction browsing history and fruit phenotype, χ^2^ = 4.65, *p* = 0.03; April: χ^2^ = 19.03, *p* < 0.0001; May: χ^2^ = 10.83, *p* = 0.001). Qualitatively, most observers easily distinguished plants from exposed as compared to naïve populations by their smaller size and decumbent habit.

Plants from historically exposed populations often formed rosettes by allocating more energy to outward than upward growth compared to plants from naïve populations (Figure 1c; Appendix S1; Day 55: χ^2^ = 24.72, *p* < 0.0001; Day 119: χ^2^ = 9.20, *p* = 0.002; Day 192: χ^2^ = 80.35, *p* < 0.0001). However, the largest differences in shape occurred at Day 192, when plants from naïve populations expressed H:W ratios 60% greater than those from historically exposed populations on average (Figure 1c). Variation in H:W ratio at day 192 (i.e. March) was also related to fruit phenotype because plants bearing wingless fruits invested more in outward than upright growth, yielding H:W ratios ~95% those of plants bearing winged fruits (Figure 1c; χ^2^ = 5.61, *p* = 0.02).

Plants from historically exposed populations produced almost twice as many branches below 10 cm height (8.0 ± 0.4) than plants from naïve populations on average (4.3 ± 0.2; Appendix S1; χ^2^ = 37.09, *p* = 0.001). A two‐way interaction of browsing history and fruit phenotype (χ^2^ = 6.93, *p* = 0.008) and post hoc contrasts confirmed that exposed populations and plants bearing wingless fruits both had more had more branches below 10 cm than other groups, even though plants from exposed populations produced slightly fewer branches in total (12.7 ± 0.7 and 14.0 ± 0.7, exposed and naïve, respectively; Appendix S1; χ^2^ = 4.99, *p* = 0.03). Similarly, the height of the lowest branch on plants from historically naïve populations was 3.2 times higher than on plants from exposed populations (3.8 ± 0.3 vs 1.20 ± 0.21 cm, respectively; Kruskal–Wallis, χ^2^ = 218.75, df = 3, *p* < 0.001).

Plants from historically naïve populations developed buds and flowered earlier than plants from historically exposed populations, as did plants bearing winged as opposed to wingless fruits (Appendix S1; browsing history: χ^2^ = 117.32, *p* < 0.0001; fruit phenotype: χ^2^ = 75.65, *p* < 0.0001; interaction browsing history and fruit phenotype: χ^2^ = 19.27, *p* < 0.0001). For example, 62.4% of plants bearing winged fruits and from historically naïve populations developed buds by Day 192 (i.e. March) and 19.6% had flowered versus 30.1% and 13.0% of plants from exposed populations (Appendix S1).

### 3.3 Intraspecific competition and trait values

**FIGURE 2:**
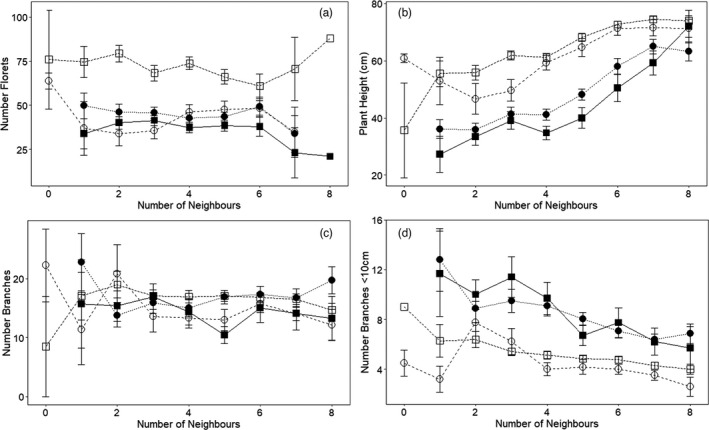
Plant morphology varied with increased number of neighbours by increasing plant height and the height of branches, but at a cost of reduced fecundity. Fecundity (estimated number of florets; (a), plant height (b), total number of branches (c) and number of branches below 10 cm (d) in May, showing mean ± standard error of raw data. Open and closed symbols represent historically naïve and exposed populations, respectively. Squares and circles represent winged and wingless fruits, respectively. Only historically naïve populations had focal individuals with 0 neighbours (n = 6)

Grouped by origin and fruit phenotype, plant height increased by 115–285% as the number of neighbours increased from zero to eight (Figure 2b; χ^2^ = 6.36, *p* = 0.01; Appendix S1). Height increases were most evident in historically exposed populations, which grew to ~25 to 35 cm with one adjacent neighbour, but to ~75 cm with eight neighbours, roughly matching the height of naïve plants at flowering (Figure 2b; post hoc tests indicated historically naïve and exposed populations differed in height, *p* < 0.0001, with no influence of fruit phenotype, *p* > 0.125). With few adjacent neighbours, plants from historically naïve populations were two to three times taller than plants from exposed populations on average (Figure 2b). The height of focal plants also increased weakly with the mean height of neighbours (χ^2^ = 3.64, *p* = 0.06; two‐way interaction between number of neighbours and mean height of neighbours, χ^2^ = 7.71, *p* = 0.005; Appendix S1). By contrast, H:W ratio (day 192) was unrelated to the number of adjacent neighbours (χ^2^ = 0.01, *p* = 0.75; Appendix S1). Fruit phenotype had no added influence plant height (χ^2^ = 0.23, *p* = 0.63) as neighbours increased, but appeared to influence H:W ratio weakly (χ^2^ = 3.60, *p* = 0.06).

Plants from historically naïve populations had about half as many branches below 10 cm as plants from historically exposed populations (browsing history: χ^2^ = 30.53, *p* < 0.0001; post hoc tests, *p* < 0.0001; other groups, *p* > 0.0125), with no influence of fruit phenotype (χ^2^ = 0.86, *p* = 0.35; post hoc tests, *p* = 0.30). However, the number of branches below 10 cm declined by ~50% as neighbours increased from zero to eight in all groups (Figure 2d; χ^2^ = 6.51, *p* = 0.01; Appendix S1), independent of the total number of branches produced, and was similar for all groups by history and phenotype (Figure 2c; browsing history: χ^2^ = 0.21, *p* =0.64; fruit phenotype: χ^2^ = 0.27, *p* = 0.60; Appendix S1).

Fecundity increased linearly with flowering height over all groups (florets / cm: 1.07 ± 0.04, β ± se, t = 28.42, df = 997, *p* < 0.0001), but was two to three times greater in plants bearing winged fruits (χ2 = 15.57, *p* < 0.0001) and from historically naïve populations (χ2 = 103.26, *p* < 0.0001; interaction origin X fruit phenotype, χ2 = 94.30, *p* < 0.0001) than in other groups (Figure 2a; Appendix S1; post hoc tests: this group versus other, *p* < 0.0001; other comparisons, *p* > 0.0125). By contrast, fecundity was unrelated to the number of neighbours (Figure 2b; χ2 = 0.37, *p* = 0.54), fraction from naïve populations (χ2 = 1.70, *p* = 0.19), or their average height (χ2 = 0.24, *p* = 0.62; Appendix S1).

Plasticity in flowering height given competition was much higher in naïve plants bearing wingless fruits (530%) than in naïve‐winged (227%), exposed‐wingless (159%) or exposed‐winged plants (120%; Appendix S1). However, contrary to our prediction that plants investing more in upright growth in response to competition might reduce fecundity, fecundity and plasticity were positively related over all plants (r = 0.66, n = 999, *p* < 0.0001, Appendix S1), but increased faster per centimetre increase in height in naïve plants bearing winged fruits (0.53 ± 0.04) than exposed‐wingless plants (0.43 ± 0.03) or exposed‐winged and naïve‐wingless plants (0.30 ± 0.03, each case; Appendix S1).

## 4 DISCUSSION

Genetic differentiation and intraspecific competition each affected plant size and shape in *P*. *congesta* populations historically exposed or naïve to browsing ungulates when grown in common gardens without them. Population‐level differences in traits shown previously to reflect local adaptation to the presence or absence of browsing ungulates in nature (Skaien & Arcese, 2018, 2020) were accentuated in the gardens described here (Figure 1). Plasticity in flowering height given competition was two to five times higher in naïve than exposed plants and related positively to fecundity in all groups (Appendix S1), contrary to our prediction that fecundity might decline as plasticity increased. These results broadly support the hypothesis that context‐dependent variation in natural selection drives local adaptation in *P*. *congesta* populations by affecting success in competition versus resistance or tolerance to browsing in the absence versus presence of ungulates, respectively. But our results also reveal substantial plasticity in plant size and shape within populations which could enhance individual fitness and the persistence of populations subject to temporal variation in the occurrence or abundance browsing ungulates (*cf* Hendry, 2015).

High evolvabilities in plant height, shape and branch position imply that differences in traits as large as those observed in our gardens could arise in 2–18 generations in nature (Table 1; Skaien & Arcese, 2020). These findings extend Carey’s (1983) demonstration of rapid adaptation in *P*. *congesta* in response to artificial selection on plant height which led to changes of +150% and −50% in five generations. Similar examples of rapid adaptation are reported from insular lizard (20 generations; Stuart et al., 2014), bird (22 years; Grant & Grant, 2006) and plant populations (8 years; Ågren et al., 2013). Our results advance understanding of the mechanisms underlying local adaptation in *P*. *congesta* populations by providing a particularly detailed picture of the selective factors at play over micro‐geographic to landscape scales.

Specifically, our results support the hypothesis that spatial heterogeneity in natural selection drives local adaptation in life history, phenotype and fitness in insular populations of *P*. *congesta* in ways that are likely to enhance the persistence of regional metapopulations (*cf* Skaien & Arcese, 2018). For example, moderate to high V_a_, h^2^, and evolvabilities in locally adapted traits affecting fitness indicate a substantial capacity for rapid adaptation in response to changes in the direction of selection associated with the colonization or extirpation of browsing ungulates. In the presence of ungulates, selection favoured shorter plants, more basal branches, bolting later and wingless fruits (Skaien & Arcese, 2018, 2020). By contrast, plants from populations without resident ungulates emphasized upright growth, earlier bolting, fewer basal branches and winged fruits (this study, Skaien & Arcese, 2018, 2020). Because directional selection can be expected to exhaust genetic variation in traits affecting fitness in the absence of gene flow, the patterns we describe are consistent with the idea that historic and/or contemporary gene flow helps to maintain adaptive capacity in the locally adapted populations of *P*. *congesta* we studied by maintaining migration‐selection balance (e.g. Hendry et al., 2018; Nosil et al., 2009; Shaw, 2019; Wright, 1982; Yeaman & Whitlock, 2011).

Historical accounts and our prior studies confirm that variation in the abundance and occurrence of browsing ungulates has been substantial over the last two centuries as a result of human influence and the colonization‐extinction dynamics of ungulate populations in the San Juan and Gulf Island archipelagos (e.g. Arcese et al., 2014; Bennett & Arcese, 2013; Best & Arcese, 2008; Gonzales & Arcese, 2008; Martin et al., 2011). For example, conditions described by European explorers in the late 1700s suggest that an ungulate eruption followed the decimation of Indigenous Peoples and eradication of large carnivores. In contrast, by the late 1800s, livestock introductions and over‐hunting nearly eliminated native ungulates before the imposition of hunting bans after 1970, which have again led to hyper‐abundant deer populations and the decimation of native birds and many species of palatable plants relied on by Indigenous Peoples prior to colonization (e.g. Arcese et al., 2014; Gonzales & Arcese, 2008; MacDougall et al., 2004; Martin et al., 2011). Given this history, we suggest that the evolutionary forces and features of insular *P*. *congesta* populations exemplified in our studies also indicate that contemporary gene flow has the potential to rescue insular populations adapted to intense competition in the absence of browsers but now declining after colonization by ungulates. Maintaining such processes should enhance the persistence of insular populations subject to rapid environmental change, particularly those with a limited capacity for rapid adaptation (e.g. Bell, 2017; Cronk, 2016; Hendry et al., 2018; Shaw, 2019).

*Plectritis congesta* grown from populations historically exposed to browsers were 28 to 40% shorter (Figure 1a,b), formed rosettes more often (Figure 1c), flowered later (Appendix S1) and produced twice as many branches within 10 cm of the ground (Figure 2d) than plants from naïve populations. These patterns are similar to those observed in *Brassica rapa* (Ramos & Schiestl, 2020) and numerous other studies of plant responses to ungulate herbivores (Diaz et al., 2006). In addition to being shorter on average, *P*. *congesta* from populations exposed to browsers were only 30–50% as fecund as plants from naïve populations in our common gardens; differences that were accentuated as competition increased (Figure 2a). These results support our prediction that the progeny of *P*. *congesta* historically exposed to browsing are likely to be at a selective disadvantage upon their immigration or transplantation into *P*. *congesta* populations naïve to browsers.

A capacity for plastic responses to increased competition could enhance the resilience of populations subject to temporal variation in competition linked to the extinction‐colonization dynamics of browsing ungulates. For example, plasticity in flowering height may enhance fitness by allowing plants to take advantage of positive conditions for growth which arise as a consequence of temporal or spatial variation in intra‐ or interspecific competition, or soil depth, nutrients or moisture at a site (Skaien & Arcese, 2018; *cf* Carey & Ganders, 1980). However, because taller plants with larger (typically winged) inflorescences survived poorly and expressed lower relative fitness than diminutive plants with smaller inflorescences (typically wingless) where browsers were common (Skaien & Arcese, 2020), we suggest that plastic increases in height in response to competition and/or growing conditions may be selected against in some populations. Our observation that plasticity given competition was higher in naïve than exposed populations on average, and somewhat higher in plants bearing wingless fruits (Appendix S1), suggests that experiment characterizations of selection on plasticity are warranted. Similar context‐dependent trade‐offs in phenotypic expression and fitness are reported between plant height, herbivory and competition for light and pollinators in *Tithonia tubaeformis* (Boege, 2010) and *P*. *farinosa* (Ågren et al., 2013), and for temperature tolerance in *Clarkia pulchella* (Bontrager & Angert, 2018) and *Escherichia coli* (Bennett & Lenski, 2007), and appear widespread in plant populations expressing a variety of locally adapted traits (e.g. Lucas‐Barbosa, 2019; Züst & Agrawal, 2017; Ramos & Schiestl, 2019; López‐Goldar & Agrawal, 2021).

*Plectritis congesta* bearing winged fruits and originating from naïve populations were 200 to 300% more fecund than all others grouped by fruit phenotype and browsing history (Figure 2a). Plants bearing winged fruits also bolted and flowered earlier than plants bearing wingless fruits (Figure 1a,b; Appendix S1), and produced larger inflorescences in experimental gardens open to and protected from deer (Skaien & Arcese, 2020; this study). These observations parallel the results of surveys of 285 *P*. *congesta* populations at 77 island and 44 mainland sites, which led to our hypothesis that directional selection favours plants bearing winged versus wingless fruits in the absence versus presence of browsers, respectively (Skaien & Arcese, 2018). Limited support for the hypothesis that selection acts directly on fruit phenotype include pilot studies suggesting that wingless fruits pass more readily through ruminant guts than winged fruits, fall more readily from inflorescences that are mechanically disturbed, and disperse slightly shorter distances than winged fruits when exposed to wind speeds typical of June to July, when *P*. *congesta* fruits typically mature in our region (our unpublished results). However, it is also plausible that selection for winged fruits in *P*. *congesta* populations naïve to browsing ungulates arises mainly due to natural selection on loci influencing success in competition, plasticity, height, inflorescence size and/or blooming phenology via genetic linkage (*cf* Carey & Ganders, 1980; Agrawal et al., 2012; Ramos & Schiestl, 2019; this study).

We also observed substantial V_p_ in three locally adapted traits in *P*. *congesta* (CV_p_ = 38 to 158; Table 1), due in part to variation in intraspecific competition (Figure 2). Carey and Ganders (1980) also showed that *P*. *congesta* grew taller and more branches in warm‐wet versus cool‐dry environmental chambers, reflecting landscape‐level correlations between climate and plant height reported across 285 sites in the San Juan and Gulf Island archipelagos (Skaien & Arcese, 2018). Gould et al. (2014) also reported substantial genetic differentiation and plasticity in response to variation in soil type, temperature and precipitation on phenotype and fitness in *Clarkia* xantiana, and many studies report declines in fecundity in response to competition as an outcome of trade‐offs in the allocation of resources to herbivore defence versus success in competition (*e*.*g*. Ballare, 2014; de Vries et al., 2019; Züst & Agrawal, 2017). Although more detailed studies will be required to explicate the mechanisms involved, our demonstration of marked plasticity in plant height, fecundity and shape in *P*. *congesta* in response to variation in local competition and the abiotic environment, and its dependence on browsing history and fruit phenotype, indicates that studies which fail to account for such factors will be of limited value when attempting to predict the performance of *P*. *congesta* in nature.

Although our current results imply that sufficient genetic variation exists among the populations we studied to facilitate rapid adaptation to variation the colonization–extinction dynamics of browsing ungulates, more work is needed to estimate adaptive capacity in isolated populations subject to directional selection, given a potential for canalization in developmental trajectory, reduced plasticity and the potential exhaustion of genetic variation in traits affecting fitness (e.g. Fisher, 1930; Hendry, 2015; Bell, 2017, Shaw, 2019). In particular, we predict that populations of *P*. *congesta* naïve to browsers and genetically isolated from populations adapted to browsing will be prone to extirpation if colonized by browsers in the absence of spatial refuges from herbivory (e.g. Skaien & Arcese, 2020).

Despite broad consistency in our results with respect to population differentiation, selection and local adaptation, we cannot rule out an influence of maternal effects on our results, which may facilitate adaptation via ‘transgenerational plasticity’ (Agrawal et al., 1999; Galloway & Etterson, 2007; McIntyre & Strauss, 2014). Mechanisms underlying such effects include epigenetic changes passed from mother to offspring and maternal contributions to propagule size, nutrition or cytoplasm (Mousseau & Fox, 1998). Such mechanisms have been shown to influence anti‐herbivore defence mechanisms in radishes (*R*. *raphanistrum*; Agrawal, 2002), Arabidopsis (*A*. *thaliana*) and tomatoes (*S*. *lycopersicum*; Rasmann et al., 2012). Although we attempted to minimize bias due to maternal effects by accounting statistically for population and family of origin, our inability to quantify such affects could have inflated our estimates of V_a_ and heritability (Lynch & Walsh, 1998; Maniatis & Pollott, 2002; Willmore et al., 2006). Maternal effects can also reduce V_p_, drive canalization in homogenous environments, or lead to correlations between maternal phenotype and environment (Kuijper & Hoyle, 2015). Our results offer a sound point of departure from which to quantify the potential effects of maternal environment on trait expression, plasticity and adaptive capacity in *P*. *congesta*.

Environmental change currently threatens the persistence of many species globally (e.g. Allan et al., 2019; Rosenberg et al., 2019), but a growing number of empirical studies indicate that a number of species display some capacity for rapid adaption in response to change (e.g. Ågren et al., 2013; Bontrager & Angert, 2018; Grant et al., 2017; Matz et al., 2018; Stuart et al., 2014; Visty et al., 2018; Walsh et al., 2019; this study). Understanding the capacity of populations to adapt to environmental change should help conservation planners prioritize populations and/or inform actions to enhance the capacity of local populations to adapt to change by replenishing genetic variation in populations restoring dispersal corridors or augmenting variation via assisted migration (e.g. Aitken & Whitlock, 2013; Olivieri et al., 2016; Rice & Emery, 2003; Shaw, 2019; Weeks et al., 2011). Characterizing the eco‐evolutionary dynamics of species and their potential for rapid adaptation should improve conservation practice by elucidating the mechanisms and pace at which populations can be expected to respond to environmental change in nature and explicating the effects of curtailing or accentuating gene flow among locally adapted, insular populations (*cf* Martin et al., 2013; Bell, 2017; Hendry et al., 2018; Shaw, 2019).

## CONFLICT OF INTEREST

The authors declare no conflict of interest.

## Supporting information

Appendix S1

## Data Availability

Data will be archived in the Dryad Digital Repository (http://dx.doi.org/) upon final acceptance and remain openly available in [repository name] at http://doi.org/doi[], reference number [].
