# Supplementary material for: On the capacity for rapid adaptation and plastic responses to herbivory and intraspecific competition in insular populations of Plectritis congesta
Source: Evol Appl. 2022 Apr 8;15(5):804–16. doi: 10.1111/eva.13371 (PMC9108306; doi:10.1111/eva.13371)
Supplement: Supplementary file 1 — Appendix S1 [file EVA-15-804-s001.docx]

**Appendices**

Sampling Locations and Fraction of Plants Bearing Either Fruit Phenotype in Populations Sown in Totem Field

**Table A1.1** Names of 20 source populations collected in 2005 and planted into the 2006-07 common garden protected from ungulate browsers in Totem Field, UBC, percentage of plants in native populations bearing wingless fruits, and number of fruits collected from each by fruit phenotype (see Methods). These populations were pooled and not in maternal family units.

| Population | % Plants with Wingless Fruits | # of Fruits (wingless, winged) |
| --- | --- | --- |
| Historically Exposed (With Deer) |  |  |
| Coal Island | 100% | 30, 0 |
| Georgeson | 85% | 11, 11 |
| Moresby Island | 98% | 11, 11 |
| Piers Island | 90% | 22, 22 |
| Prevost Island | 99% | 44, 0 |
| Historically Naïve (Without Deer) |  |  |
| Anniversary Island | 32% | 11, 11 |
| Blunden Island | 5% | 7, 18 |
| Brackman Island | 22% | 12, 11 |
| Bright Island | 20% | 21, 22 |
| Canoe Cove | 28% | 22, 22 |
| Clive Island | 4% | 7, 15 |
| East Chanel Island | 1% | 0, 44 |
| West Chanel Island | 1% | 0, 44 |
| Hawkins Islets | 1% | 22, 22 |
| Owl Island | 9% | 22, 22 |
| Middle Pellow Island | 7% | 22, 22 |
| West Pellow Island | 3% | 11, 11 |
| Reay Island | 3% | 22, 22 |
| Rubly Island | 14% | 22, 22 |
| Sallas Rocks | 5% | 0, 44 |

**Table A1.2** Names of 17 source populations collected in 2006 and planted into the 2006-07 common garden protected from ungulate browsers in Totem Field, UBC, percentage of plants in native populations bearing wingless fruits, and the number of families collected from each by fruit phenotype (see Methods).

| Population | % Plants with Wingless Fruits | Families (wingless, winged) |
| --- | --- | --- |
| Historically Exposed (With Deer) |  |  |
| Georgeson | 85% | 7, 5 |
| Little Samuel Island | 80% | 7, 5 |
| Moresby Island | 98% | 10, 0 |
| Crow’s Nest (Salt Spring Island) | 94% | 10, 2 |
| Historically Naïve (Without Deer) |  |  |
| North Ada Island | 3% | 4, 3 |
| South Ada Island | 17% | 3, 4 |
| Anniversary Island | 32% | 4, 8 |
| Belle Island | 50% | 5, 7 |
| Blunden Island | 5% | 0, 12 |
| Brackman Island | 22% | 1, 11 |
| Clive Island | 4% | 0, 12 |
| East Dock Island | 3% | 1, 7 |
| West Dock Island | 4% | 0, 10 |
| East Pellow Island | 2% | 2, 8 |
| West Pellow Island | 3% | 0, 12 |
| Ruth Island | 5% | 0, 10 |
| Shell Island | 3% | 3, 8 |

**Table A1.3** Names of 21 source populations collected in 2015 and planted into the 2015-16 common garden protected from ungulate browsers in Totem Field, UBC, percentage of plants in native populations bearing wingless fruits, and the number of families collected from each by fruit phenotype (see Methods).

| Population | % Plants with Wingless Fruits | Families (wingless, winged) |
| --- | --- | --- |
| Historically Exposed (With Deer) |  |  |
| Dean Park (Vancouver Island) | 93% | 6, 6 |
| Galiano Island | 54% | 6, 7 |
| Mayne Island | 93% | 7, 5 |
| Moresby Island | 98% | 6, 6 |
| Pender Island | 94% | 6, 6 |
| Piers Island | 90% | 6, 6 |
| Prevost Island | 99% | 6, 6 |
| AVNR (Salt Spring Island) | 93% | 6, 6 |
| Sansum Narrows (Salt Spring  Island) | 87% | 8, 4 |
| Schooner Cove | 56% | 9, 3 |
| Historically Naïve (Without Deer) |  |  |
| Canoe Cove | 28% | 6, 6 |
| Chades Island | 3% | 6, 6 |
| Chanel Islands | 1% | 5, 7 |
| Clive Island | 4% | 6, 6 |
| Grace Islet | 2% | 2, 10 |
| Isabella Island | 3% | 0, 12 |
| Musgrave Island | 1% | 6, 6 |
| Owl Island | 9% | 6, 6 |
| Port Washington Island | 1% | 1, 11 |
| Shell Island | 3% | 3, 9 |
| South Winchelsea Island | 7% | 3, 9 |

: Florets Given Inflorescence Volume and Height Given Competition

We use normal regression to estimate the number of florets produced per plant given inflorescence volume in 167 plants described in detail using data from an exclosed, experimental population on Sidney Island (see Skaien & Arcese 2020). Inflorescence volume was estimated as an ellipsoid (V = (4/3)*abc, where a was the distance from the midpoint to the top of the inflorescence, and b and c were the distance from the midpoint to the outer edge of the inflorescence). Random effects included in the model were ‘population’ and ‘substrate (coded as primarily grass, primarily moss, or a mixture of these; see Skaien & Arcese 2020; fitted regression, R­_adj_^2^ = 0.50).

$Predicted Florets=exp(3.0305-0.1343\left( deer=absent \right)+0.1778\left( fruit=wingless \right)-0.1476\left( deer=absent, fruit=wingless \right)+0.3391\left( LogInflV \right)+0.0994*\left( logInflV \right)\left( deer=absent \right)+0.0365*\left( logInflV \right)\left( fruit=wingless \right)-0.1297*(LogInflV)(deer=absent)(fruit=wingless)$

$$Predicted Florets:Exposed Wingless = exp(3.2083+0.3756*(logINFLV)$$

$$Predicted Florets:Exposed Winged = exp(3.0305+0.3391*(logINFLV)$$

$$Predicted Florets:Naive Wingless = exp(2.9264+0.3453*(logINFLV)$$

$$Predicted Florets:Naive Winged = exp(2.8962+0.4385*(logINFLV)$$

*Height Given Competition*

We expressed plasticity in height given competition (CV_Ht_ | C) as the coefficient of variation (CV) of observed plant height in May minus its expected height in the absence of competition, e.g., Plasticity | competition = ((ht_May_ – ht_exp_) / ht_exp_) * 100 (data approximately normally distributed; *cf* Arnold et al., 2019; Valladares et al., 2006). Expected height was estimated by regressing May height on factors also used the characterize competition in Figure 2 and Tables A8 – A12 below using a general linear mixed model (Gausian distribution, untransformed fixed effects = *Browsing History, Fruit Phenotype of Surviving Plant, Proportion of Neighbours from Historically Exposed Populations, Number of Living Neighbours, Mean Height of Neighbouring Plants*, random effect = *bed*). We then calculated Plasticity | competition for each plant by expected phenotype to test our predictions with respect to fecundity, competition, and plasticity in May height, given browsing history and fruit phenotype (see Introduction). The graphical results from the latter analyses for historically exposed (a) and naïve populations (b), equations for regressions shown, and estimated plasticity | competition for each group listed below.

b

a

CV_Ht_ | C *and its Regression on fecundity by group*

*Exposed Winged*: CV_Ht_|C = 120.07. Fecundity = 53.643 (1.167) + 0.308 (0.027) * CV_Ht_ | Competition, Rsq_adj_ = 0.45, F_1,147_ = 130.63, P< 0.0001.

*Exposed Wingless:* CV_Ht_ | C = 158.96. Fecundity = 64.677 (1.109) + 0.432 (0.028) * CV_Ht_ | Competition, Rsq_adj_ = 0.43, F_1,303_ = 233.45, P< 0.0001.

*Naïve Winged*: CV_Ht_ | C = 226.75. Fecundity = 73.044 (1.366) + 0.526 (0.035)* CV_Ht_ | Competition, Rsq_adj_ = 0.35,F_1 ,411_ = 221.76, P< 0.0001.

*Naive, Wingless*: CV_Ht_ | C = 529.9. Fecundity = 48.457 (1.277) + 0.305 (0.030)*Ht| Competition, Rsq_adj_ = 0.46, F_1,120_ = 103.73, P< 0.0001

: ANOVA Results for Plant Height in the Totem Field Common Gardens

**Table A3.1** ANOVA results for the model predicting plant height at day 55 in the Totem Field common gardens (2006-07, 2015-16), including Wald’s Chi-Square and p-values for fixed effect variables and interactions between variables, using Type III tests. Bold p-values denote statistical significance.

| **Fixed Effects Variable or Interaction** | **Df** | **χ^2^** | **p-value** |
| --- | --- | --- | --- |
| **Fixed Effects Variable** |  |  |  |
| Intercept | 1 | 36.36 | **<0.0001** |
| Browsing History | 1 | 35.21 | **<0.0001** |
| Fruit Phenotype of Sown Fruit | 1 | 26.17 | **<0.0001** |
| **Two-way Interactions of Fixed Effects** |  |  |  |
| Browsing History* Fruit Phenotype of Sown Fruit | 1 | 0.60 | 0.44 |

**Table A3.2** Variance accounted for by random effects variables in the model predicting plant height at day 55 in the Totem Field common gardens (2006-07, 2015-16).

| **Random Effects Variable** | **Variance** | **SD** |
| --- | --- | --- |
| Garden Year | 8.5 x 10^-3^ | 9.2 x 10^-2^ |
| Garden Year/Bed ID | 7.5 x 10^-3^ | 8.6 x 10^-2^ |
| Population | 0.13 | 0.36 |
| Residual | 0.49 | 6.0 x 10^-3^ |

**Table A3.3** ANOVA results for the model predicting plant height at day 119 in the Totem Field common gardens (2006-07, 2015-16), including Wald’s Chi-Square and p-values for fixed effect variables and interactions between variables, using Type III tests. Bold p-values denote statistical significance.

| **Fixed Effects Variable or Interaction** | **Df** | **χ^2^** | **p-value** |
| --- | --- | --- | --- |
| **Fixed Effects Variable** |  |  |  |
| Intercept | 1 | 71.92 | **<0.0001** |
| Browsing History | 1 | 61.08 | **<0.0001** |
| Fruit Phenotype of Sown Fruit | 1 | 24.72 | **<0.0001** |
| **Two-way Interactions of Fixed Effects** |  |  |  |
| Browsing History* Fruit Phenotype of Sown Fruit | 1 | 3.32 | 0.07 |

**Table A3.4** Variance accounted for by random effects variables in the model predicting plant height at day 119 in the Totem Field common gardens (2006-07, 2015-16).

| **Random Effects Variable** | **Variance** | **SD** |
| --- | --- | --- |
| Garden Year | 9.4 x 10^-3^ | 9.7 x 10^-2^ |
| Garden Year/Bed ID | 5.6 x 10^-3^ | 7.5 x 10^-2^ |
| Population | 3.9 x 10^-2^ | 0.20 |
| Residual | 0.39 | 4.7 x 10^-3^ |

**Table A3.5** ANOVA results for the model predicting plant height at day 192 in the Totem Field common gardens (2006-07, 2015-16), including Wald’s Chi-Square and p-values for fixed effect variables and interactions between variables, using Type III tests. Bold p-values denote statistical significance.

| **Fixed Effects Variable or Interaction** | **Df** | **χ^2^** | **p-value** |
| --- | --- | --- | --- |
| **Fixed Effects Variable** |  |  |  |
| Intercept | 1 | 979.47 | **<0.0001** |
| Browsing History | 1 | 56.88 | **<0.0001** |
| Fruit Phenotype of Sown Fruit | 1 | 51.52 | **<0.0001** |
| **Two-way Interactions of Fixed Effects** |  |  |  |
| Browsing History* Fruit Phenotype of Sown Fruit | 1 | 4.65 | **0.03** |

**Table A3.6** Variance accounted for by random effects variables in the model predicting plant height at day 192 in the Totem Field common gardens (2006-07, 2015-16).

| **Random Effects Variable** | **Variance** | **SD** |
| --- | --- | --- |
| Garden Year | 2.4 x 10^-3^ | 4.9 x 10^-2^ |
| Garden Year/Bed ID | 4.9 x 10^-3^ | 6.9 x 10^-2^ |
| Population | 8.4 x 10^-2^ | 0.29 |
| Residual | 0.41 | 5.0 x 10^-2^ |

**Table A3.7** ANOVA results for the model predicting plant height in April in the Totem Field common gardens (2006-07, 2015-16), including Wald’s Chi-Square and p-values for fixed effect variables and interactions between variables, using Type III tests. Bold p-values denote statistical significance.

| **Fixed Effects Variable or Interaction** | **Df** | **χ^2^** | **p-value** |
| --- | --- | --- | --- |
| **Fixed Effects Variable** |  |  |  |
| Intercept | 1 | 191.88 | **<0.0001** |
| Browsing History | 1 | 17.87 | **<0.0001** |
| Fruit Phenotype of Sown Fruit | 1 | 19.03 | **<0.0001** |
| **Two-way Interactions of Fixed Effects** |  |  |  |
| Browsing History* Fruit Phenotype of Sown Fruit | 1 | 0.01 | 0.92 |

**Table A3.8** Variance accounted for by random effects variables in the model predicting plant height in April in the Totem Field common gardens (2006-07, 2015-16).

| **Random Effects Variable** | **Variance** | **SD** |
| --- | --- | --- |
| Garden Year | 0.16 | 0.39 |
| Garden Year/Bed ID | 1.4 x 10^-3^ | 3.7 x 10^-2^ |
| Population | 5.9 x 10^-2^ | 0.24 |
| Residual | 0.36 | 4.4 x 10^-3^ |

**Table A3.9** ANOVA results for the model predicting plant height in May in the Totem Field common gardens (2006-07, 2015-16), including Wald’s Chi-Square and p-values for fixed effect variables and interactions between variables, using Type III tests. Bold p-values denote statistical significance.

| **Fixed Effects Variable or Interaction** | **Df** | **χ^2^** | **p-value** |
| --- | --- | --- | --- |
| **Fixed Effects Variable** |  |  |  |
| Intercept | 1 | 650.50 | **<0.0001** |
| Browsing History | 1 | 20.51 | **<0.0001** |
| Fruit Phenotype of Sown Fruit | 1 | 10.83 | **0.001** |
| **Two-way Interactions of Fixed Effects** |  |  |  |
| Browsing History* Fruit Phenotype of Sown Fruit | 1 | 0.53 | 0.47 |

**Table A3.10** Variance accounted for by random effects variables in the model predicting plant height in May in the Totem Field common gardens (2006-07, 2015-16).

| **Random Effects Variable** | **Variance** | **SD** |
| --- | --- | --- |
| Garden Year | 4.8 x 10^-2^ | 0.22 |
| Garden Year/Bed ID | 4.2 x 10^-3^ | 6.5 x 10^-2^ |
| Population | 3.4 x 10^-2^ | 6.5 x 10^-2^ |
| Residual | 0.36 | 4.3 x 10^-3^ |

: ANOVA Results for Growth Form (H:W Ratio) in the Totem Field Common Gardens

**Table A4.1** ANOVA results for the model predicting growth form (measured as the height to width ratio, H:W ratio, natural log transformed) at day 55 in the Totem Field common gardens (2006-07, 2015-16), including Wald’s Chi-Square and p-values for fixed effect variables and interactions between variables, using Type III tests. Bold p-values denote statistical significance.

| **Fixed Effects Variable or Interaction** | **Df** | **χ^2^** | **p-value** |
| --- | --- | --- | --- |
| **Fixed Effects Variable** |  |  |  |
| Intercept | 1 | 65.10 | **<0.0001** |
| Browsing History | 1 | 24.72 | **<0.0001** |
| Fruit Phenotype of Sown Fruit | 1 | 0.13 | 0.72 |
| **Two-way Interactions of Fixed Effects** |  |  |  |
| Browsing History* Fruit Phenotype of Sown Fruit | 1 | 0.94 | 0.33 |

**Table A4.2** Variance accounted for by random effects variables in the model predicting growth form (measured as the height to width ratio, H:W ratio) at day 55 in the Totem Field common gardens (2006-07, 2015-16).

| **Random Effects Variable** | **Variance** | **SD** |
| --- | --- | --- |
| Garden Year | 1.8 x 10^-2^ | 0.13 |
| Garden Year/Bed ID | 3.0 x 10^-3^ | 5.5 x 10^-2^ |
| Population | 7.6 x 10^-2^ | 0.28 |
| Residual | 0.46 | 5.6 x 10^-3^ |

**Table A4.3** ANOVA results for the model predicting growth form (measured as the height to width ratio, H:W ratio, natural log transformed) at day 119 in the Totem Field common gardens (2006-07, 2015-16), including Wald’s Chi-Square and p-values for fixed effect variables and interactions between variables, using Type III tests. Bold p-values denote statistical significance.

| **Fixed Effects Variable or Interaction** | **Df** | **χ^2^** | **p-value** |
| --- | --- | --- | --- |
| **Fixed Effects Variable** |  |  |  |
| Intercept | 1 | 569.84 | **<0.0001** |
| Browsing History | 1 | 9.20 | **0.002** |
| Fruit Phenotype of Sown Fruit | 1 | 2.05 | 0.15 |
| **Two-way Interactions of Fixed Effects** |  |  |  |
| Browsing History* Fruit Phenotype of Sown Fruit | 1 | 1.10 | 0.29 |

**Table A4.4** Variance accounted for by random effects variables in the model predicting growth form (measured as the height to width ratio, H:W ratio) at day 119 in the Totem Field common gardens (2006-07, 2015-16).

| **Random Effects Variable** | **Variance** | **SD** |
| --- | --- | --- |
| Garden Year | 3.8 x 10^-3^ | 6.2 x 10^-2^ |
| Garden Year/Bed ID | 7.0 x 10^-3^ | 8.4 x 10^-2^ |
| Population | 1.4 x 10^-2^ | 0.12 |
| Residual | 0.35 | 4.3 x 10^-3^ |

**Table A4.5** ANOVA results for the model predicting growth form (measured as the height to width ratio, H:W ratio, natural log transformed) at day 192 in the Totem Field common gardens (2006-07, 2015-16), including Wald’s Chi-Square and p-values for fixed effect variables and interactions between variables, using Type III tests. Bold p-values denote statistical significance.

| **Fixed Effects Variable or Interaction** | **Df** | **χ^2^** | **p-value** |
| --- | --- | --- | --- |
| **Fixed Effects Variable** |  |  |  |
| Intercept | 1 | 0.85 | 0.36 |
| Browsing History | 1 | 80.35 | **<0.0001** |
| Fruit Phenotype of Sown Fruit | 1 | 5.61 | **0.02** |
| **Two-way Interactions of Fixed Effects** |  |  |  |
| Browsing History* Fruit Phenotype of Sown Fruit | 1 | 1.02 | 0.31 |

**Table A4.6** Variance accounted for by random effects variables in the model predicting growth form (measured as the height to width ratio, H:W ratio) at day 192 in the Totem Field common gardens (2006-07, 2015-16).

| **Random Effects Variable** | **Variance** | **SD** |
| --- | --- | --- |
| Garden Year | 5.4 x 10^-2^ | 0.23 |
| Garden Year/Bed ID | 8.1 x 10^-3^ | 9.0 x 10^-2^ |
| Population | 2.1 x 10^-2^ | 0.15 |
| Residual | 0.27 | 3.3 x 10^-3^ |

: ANOVA Results for the Total Number of Branches in the Totem Field Common Gardens

**Table A5.1** ANOVA results for the model predicting the total number of branches (negative binomial distribution) in the Totem Field common gardens (2006-07, 2015-16), including Wald’s Chi-Square and p-values for fixed effect variables and interactions between variables, using Type III tests. Bold p-values denote statistical significance.

| **Fixed Effects Variable or Interaction** | **Df** | **χ^2^** | **p-value** |
| --- | --- | --- | --- |
| **Fixed Effects Variable** |  |  |  |
| Intercept | 1 | 19.56 | **< 0.0001** |
| Browsing History | 1 | 0.29 | 0.78 |
| Fruit Phenotype of Surviving Plant | 1 | -3.65 | **< 0.001** |
| **Two-way Interactions of Fixed Effects** |  |  |  |
| Browsing History* Fruit Phenotype of Surviving Plant | 1 | 2.56 | **0.01** |

**Table A5.2** Variance accounted for by random effects variables in the model predicting the total number of branches (natural log transformed) in the Totem Field common gardens (2006-07, 2015-16).

| **Random Effects Variable** | **Variance** | **SD** |
| --- | --- | --- |
| Garden Year | 0.008 | 0.09 |
| Garden Year/Bed ID | 0.04 | 0.19 |
| Population | 0.03 | 0.19 |
|  |  |  |

: ANOVA Results for the Number of Branches Below 10 cm Height in the Totem Field Common Gardens

**Table A6.1** ANOVA results for the model predicting the number of branches below 10 cm of height in the Totem Field common gardens (2006-07, 2015-16), including Wald’s Chi-Square and p-values for fixed effect variables and interactions between variables, using Type III tests. Bold p-values denote statistical significance.

| **Fixed Effects Variable or Interaction** | **Df** | **χ^2^** | **p-value** |
| --- | --- | --- | --- |
| **Fixed Effects Variable** |  |  |  |
| Intercept | 1 | 610.39 | **< 0.0001** |
| Browsing History | 1 | 37.09 | **< 0.0001** |
| Fruit Phenotype of Surviving Plant | 1 | 9.24 | **0.002** |
| **Two-way Interactions of Fixed Effects** |  |  |  |
| Browsing History* Fruit Phenotype of Surviving Plant | 1 | 6.93 | **0.008** |

**Table A6.2** Variance accounted for by random effects variables in the model predicting the number of branches below 10 cm of height in the Totem Field common gardens (2006-07, 2015-16).

| **Random Effects Variable** | **Variance** | **SD** |
| --- | --- | --- |
| Garden Year | 2.0 x 10^-3^ | 4.5 x 10^-2^ |
| Garden Year/Bed ID | 5.3 x 10^-3^ | 7.3 x 10^-2^ |
| Population | 4.6 x 10^-2^ | 0.21 |
| Residual | 0.77 | 9.4 x 10^-3^ |

: ANOVA Results for Flowering Phenology in the Totem Field Common Gardens

**Table A7.1** ANOVA results for the model predicting flowering phenology in the Totem Field common gardens (2006-07, 2015-16), including Wald’s Chi-Square and p-values for fixed effect variables and interactions between variables, using Type III tests. Bold p-values denote statistical significance.

| **Fixed Effects Variable or Interaction** | **Df** | **χ^2^** | **p-value** |
| --- | --- | --- | --- |
| **Fixed Effects Variable** |  |  |  |
| Browsing History | 2 | 117.32 | **< 0.0001** |
| Fruit Phenotype of Surviving Plant | 2 | 75.65 | **< 0.0001** |
| **Two-way Interactions of Fixed Effects** |  |  |  |
| Browsing History* Fruit Phenotype of Surviving Plant | 2 | 19.27 | **< 0.0001** |

Table A7.2. Predicted percentage of plants at each developmental stage (no bud formation, buds forming or flowers present) at day 192 for historically exposed and naïve populations of both fruit phenotypes (multinomial logistic regression; Appendix 7).

|  | No Buds | Pink Buds | Blooming |
| --- | --- | --- | --- |
| Historically Exposed |  |  |  |
| Winged | 57.0% | 30.1% | 13.0% |
| Wingless | 66.1% | 27.1% | 6.8% |
| Historically Naïve |  |  |  |
| Winged | 20.1% | 62.4% | 19.6% |
| Wingless | 51.5% | 39.5% | 9.0% |

: ANCOVA Results for Fecundity Relative to Neighbour Composition in the Totem Field Common Gardens

**Table A8.1** ANCOVA results for the model predicting fecundity (number of florets) relative to neighbour composition in the Totem Field common garden (2015-16), including Wald’s Chi-Square and p-values for fixed effect variables and interactions between variables, using Type III tests. Bold p-values denote statistical significance.

| **Fixed Effects Variable or Interaction** | **Df** | **χ^2^** | **p-value** |
| --- | --- | --- | --- |
| **Fixed Effects Variable** |  |  |  |
| Browsing History | 1 | 0.17 | 0.68 |
| Fruit Phenotype of Surviving Plant | 1 | 81.73 | **< 0.0001** |
| Proportion of Neighbours from Historically Exposed Populations | 1 | 0.02 | 0.89 |
| Number of Living Neighbours | 1 | 2.06 | 0.15 |
| Mean Height of Neighbouring Plants | 1 | 0.40 | 0.53 |
| **Two-way Interactions of Fixed Effects** |  |  |  |
| Browsing History* Fruit Phenotype of Surviving Plant | 1 | 52.46 | **< 0.0001** |
| Proportion of Neighbours from Historically Exposed Populations *  Number of Living Neighbours | 1 | 0.68 | 0.41 |
| Number of Living Neighbours * Mean Height of Neighbouring  Plants | 1 | 0.52 | 0.47 |

**Table A8.2** Variance accounted for by random effects in the model predicting fecundity (number of florets) relative to neighbour composition in the Totem Field common garden (2015-16).

| **Random Effects Variable** | **Variance** | **SD** |
| --- | --- | --- |
| Bed ID | 1.6 x 10^-2^ | 0.13 |
| Population | 1.8 x 10^-3^ | 0.04 |
| Population/Family | 5.8 x 10^-3^ | 0.08 |

**Table A8.3** Estimates and modifications for each term in the model predicting fecundity (number of florets) relative to neighbour composition in the Totem Field common garden (2015-16), including independent variables and those nested in other variables, using Type III tests. Estimates are with a Poisson distribution and a log link. Intercept represents the base value for a population that is historically exposed to herbivores with winged fruits.

| **Fixed Effects Variable** | **Estimate** | **SE** | **z-value** | **p-value** |
| --- | --- | --- | --- | --- |
| Intercept | 4.07 | 0.11 | 37.74 | <0.0001 |
| Browsing History: Historically Naïve | 0.02 | 0.05 | 0.42 | 0.68 |
| Fruit Phenotype of Surviving Plant: Wingless | -0.32 | 0.04 | -9.04 | < 0.0001 |
| Proportion of Neighbours from Historically Exposed  Populations | 0.13 | 0.09 | 0.13 | 0.89 |
| Number of Living Neighbours | -0.04 | 0.03 | -1.44 | 0.15 |
| Mean Height of Neighbouring Plants | 0.001 | 0.002 | 0.63 | 0.53 |
| Browsing History: Historically Naïve * Fruit  Phenotype of Surviving Plant: Wingless | 0.36 | 0.05 | 7.24 | < 0.0001 |
| Proportion of Neighbours from Historically Exposed  Populations * Number of Living Neighbours | 0.02 | 0.03 | 0.82 | 0.41 |
| Number of Living Neighbours * Mean Height of  Neighbouring Plants | 0.00039 | 0.00 | 0.72 | 0.47 |

**Historically Exposed, plants with winged fruits:**

# of florets = exp (4.07 + 0.13(proportion of neighbours from historically exposed populations) – 0.04(# living neighbours) + 0.001(Mean height of neighbouring plants) + 0.02 (proportion of neighbours from historically exposed populations)( # living neighbours) + 0.00039 (# living neighbours)( Mean height of neighbouring plants) )

**Historically Exposed, plants with wingless fruits:**

# of florets = exp (3.75 + 0.13(proportion of neighbours from historically exposed populations) – 0.04(# living neighbours) + 0.001(Mean height of neighbouring plants) + 0.02(proportion of neighbours from historically exposed populations)( # living neighbours) + 0.00039 (# living neighbours)( Mean height of neighbouring plants) )

**Historically Naïve, plants with winged fruits:**

# of florets = exp (4.09 + 0.13(proportion of neighbours from historically exposed populations) – 0.04(# living neighbours) + 0.001(Mean height of neighbouring plants) + 0.02(proportion of neighbours from historically exposed populations)( # living neighbours) + 0.00039 (# living neighbours)( Mean height of neighbouring plants) )

**Historically Naïve, plants with wingless fruits:**

# of florets = exp (3.77+ 0.13(proportion of neighbours from historically exposed populations) – 0.04(# living neighbours) + 0.001(Mean height of neighbouring plants) + 0.02(proportion of neighbours from historically exposed populations)( # living neighbours) + 0.00039 (# living neighbours)( Mean height of neighbouring plants) )


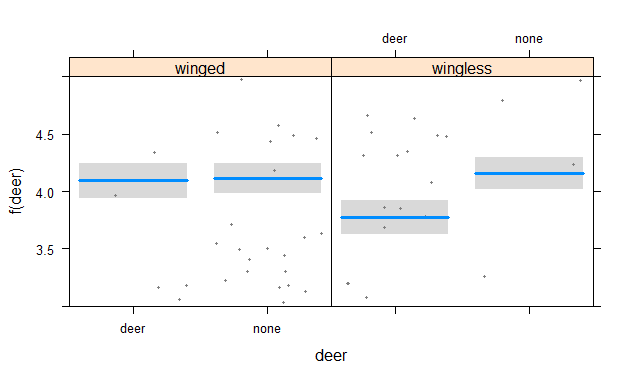


**Figure A8.1** The partial residual plot visualizing the interaction between historical exposure to browsing (populations with deer or none) and fruit phenotype for the model predicting the number of florets (as a proxy for fecundity). This plot visualizes that plants from islands with deer with wingless fruits had lower fecundity than all other groups (Browsing History* Fruit Phenotype of Surviving Plant, p < 0.0001). Plots generated using the visreg package in R.

: ANCOVA Results for Plant Height Relative to Neighbour Composition in the Totem Field Common Gardens

**Table A9.1** ANCOVA results for the model predicting plant height (cm) relative to neighbour composition in the Totem Field common garden (2015-16), including Wald’s Chi-Square and p-values for fixed effect variables and interactions between variables, using Type III tests. Bold p-values denote statistical significance.

| **Fixed Effects Variable or Interaction** | **Df** | **χ^2^** | **p-value** |
| --- | --- | --- | --- |
| **Fixed Effects Variable** |  |  |  |
| Intercept | 1 | 26.53 | **< 0.0001** |
| Browsing History | 1 | 31.48 | **< 0.0001** |
| Fruit Phenotype of Surviving Plant | 1 | 0.23 | 0.63 |
| Proportion of Neighbours from Historically Exposed Populations | 1 | 2.19 | 0.14 |
| Number of Living Neighbours | 1 | 6.36 | **0.01** |
| Mean Height of Neighbouring Plants | 1 | 3.64 | 0.06 |
| **Two-way Interactions of Fixed Effects** |  |  |  |
| Browsing History* Fruit Phenotype of Surviving Plant | 1 | 6.23 | **0.01** |
| Proportion of Neighbours from Historically Exposed Populations *  Number of Living Neighbours | 1 | 0.01 | 0.93 |
| Number of Living Neighbours * Mean Height of Neighbouring  Plants | 1 | 7.71 | **0.005** |

**Table A9.2** Variance accounted for by random effects in the model predicting plant height (cm) relative to neighbour composition in the Totem Field common garden (2015-16), including independent variables and those nested in other variables.

| **Random Effects Variable** | **Variance** | **SD** |
| --- | --- | --- |
| Garden Year | 83.85 | 9.16 |
| Garden Year/Bed ID | 1.53 | 1.24 |
| Population | 60.82 | 7.80 |
| Population/Family | 35.92 | 5.99 |
| Residual | 14.09 | 0.22 |

**Table A9.3** Estimates and modifications for each term in the model for plant height (cm) relative to neighbour composition in the Totem Field common garden (2015-16), including independent variables and those nested in other variables. Intercept represents the base value for a population that is historically exposed to herbivores with winged fruits.

| **Fixed Effects Variable** | **Estimate** | **SE** | **z-value** | **p-value** |
| --- | --- | --- | --- | --- |
| Intercept | 40.88 | 7.94 | 5.15 | < 0.0001 |
| Browsing History: Historically Naïve | 16.82 | 3.00 | 5.61 | < 0.0001 |
| Fruit Phenotype of Surviving Plant: Wingless | 0.65 | 1.35 | 0.48 | 0.63 |
| Proportion of Neighbours from Historically Exposed  Populations | 4.93 | 3.33 | 1.48 | 0.14 |
| Number of Living Neighbours | -2.00 | 0.79 | -2.52 | 0.01 |
| Mean Height of Neighbouring Plants | 0.11 | 0.06 | 1.91 | 0.06 |
| Browsing History: Historically Naïve * Fruit  Phenotype of Surviving Plant: Wingless | -4.29 | 1.72 | -2.50 | 0.01 |
| Proportion of Neighbours from Historically Exposed  Populations * Number of Living Neighbours | -0.07 | 0.73 | -0.09 | 0.93 |
| Number of Living Neighbours * Mean Height of  Neighbouring Plants | 0.03 | 0.01 | 2.78 | 0.01 |

**Historically Exposed, plants with winged fruits:**

Plant Height = 40.88 + 4.93(proportion of neighbours from historically exposed populations) – 2.00(# living neighbours) + 0.11(Mean height of neighbouring plants) – 0.07(proportion of neighbours from historically exposed populations)(# living neighbours) + 0.03 (# living neighbours)( Mean height of neighbouring plants)

**Historically Exposed, plants with wingless fruits:**

Plant Height = 41.53 + 4.93(proportion of neighbours from historically exposed populations) – 2.00(# living neighbours) + 0.11(Mean height of neighbouring plants) – 0.07(proportion of neighbours from historically exposed populations)(# living neighbours) + 0.03 (# living neighbours)( Mean height of neighbouring plants)

**Historically Naïve, plants with winged fruits:**

Plant Height = 57.70 + 4.93(proportion of neighbours from historically exposed populations) – 2.00(# living neighbours) + 0.11(Mean height of neighbouring plants) – 0.07(proportion of neighbours from historically exposed populations)(# living neighbours) + 0.03 (# living neighbours)( Mean height of neighbouring plants)

**Historically Naïve, plants with wingless fruits:**

Plant Height = 53.41 + 4.93(proportion of neighbours from historically exposed populations) – 2.00(# living neighbours) + 0.11(Mean height of neighbouring plants) – 0.07(proportion of neighbours from historically exposed populations)(# living neighbours) + 0.03 (# living neighbours)( Mean height of neighbouring plants)


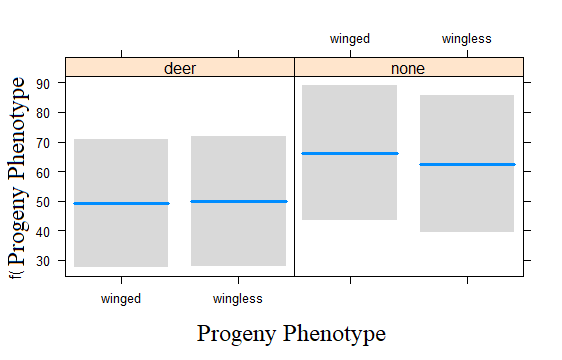

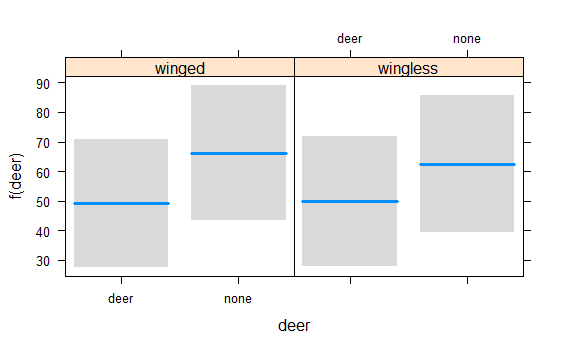


**Figure A9.1** The partial residual plot visualizing the interaction between historical exposure to browsing (populations with deer or none) and fruit phenotype for the model predicting plant height in May. This plot demonstrates that plants from populations without deer (none) were on average taller than plants from populations without deer, and that plant with winged fruits and from populations without deer were slightly taller than other groups, on average (Browsing History* Fruit Phenotype of Surviving Plant, p = 0.01). Plots generated using the visreg package in R.


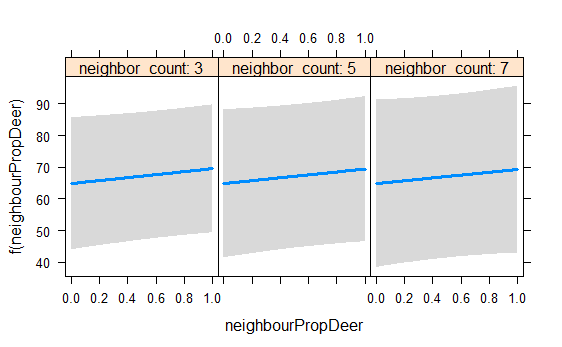


**Figure A9.2** The partial residual plot visualizing the relationship between the proportion of neighbouring plants from populations with deer, and the number of neighbours, for the model predicting plant height in May. This plot demonstrates a trend that as more plants are from populations historically exposed to herbivores, and fewer are from populations historically naïve to herbivores which tend to be taller, that the focal plant was taller ( Proportion of Neighbours from Historically Exposed Populations, p =0.14). Plots generated using the visreg package in R.


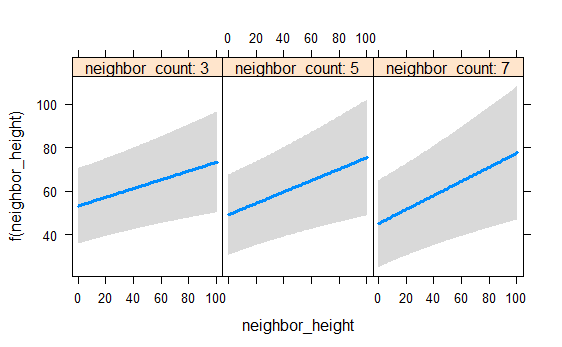


**Figure A9.3** The partial residual plot visualizing the interaction between the mean height of neighbouring plants, and the number of neighbours, for the model predicting plant height in May. This plot shows that focal plants became taller as the mean height of their neighbours increased, and that the slope of this relationship was greater with more neighbours ( Number of Living Neighbours * Mean Height of Neighbouring Plants, p =.005). Plots generated using the visreg package in R.

: ANCOVA Results for Growth Form (H:W Ratio) Relative to Neighbour Composition in the Totem Field Common Gardens

**Table A10.1** ANCOVA results for the model predicting growth form (measured as the height to width ratio, H:W ratio; natural log transformed) at day 192 relative to neighbour composition in the Totem Field common garden (2015-16), including Wald’s Chi-Square and p-values for fixed effect variables and interactions between variables, using Type III tests. Bold p-values denote statistical significance.

| **Fixed Effects Variable or Interaction** | **Df** | **χ^2^** | **p-value** |
| --- | --- | --- | --- |
| **Fixed Effects Variable** |  |  |  |
| Intercept | 1 | 12.26 | **< 0.001** |
| Browsing History | 1 | 59.49 | **< 0.0001** |
| Fruit Phenotype of Surviving Plant | 1 | 3.60 | 0.06 |
| Proportion of Neighbours from Historically Exposed Populations | 1 | 0.63 | 0.43 |
| Number of Living Neighbours | 1 | 0.10 | 0.75 |
| Mean Height of Neighbouring Plants | 1 | 1.13 | 0.29 |
| **Two-way Interactions of Fixed Effects** |  |  |  |
| Browsing History* Fruit Phenotype of Surviving Plant | 1 | 0.10 | 0.75 |
| Proportion of Neighbours from Historically Exposed Populations *  Number of Living Neighbours | 1 | 0.06 | 0.81 |
| Number of Living Neighbours * Mean Height of Neighbouring  Plants | 1 | 3.18 | 0.07 |

**Table A10.2** Variance accounted for by random effects in the model predicting for growth form (measured as the height to width ratio, H:W ratio) at day 192 relative to neighbour composition in the Totem Field common garden (2015-16).

| **Random Effects Variable** | **Variance** | **SD** |
| --- | --- | --- |
| Garden Year | 7.5 x 10^-2^ | 0.27 |
| Garden Year/Bed ID | 7.9 x 10^-3^ | 8.9 x 10^-2^ |
| Population | 2.5 x 10^-2^ | 0.16 |
| Population/Family | 9.7 x 10^-3^ | 9.9 x 10^-2^ |
| Residual | 0.23 | 3.8 x 10^-3^ |

**Table A10.3** Estimates and modifications for each term in the model for growth form (H:W Ratio; natural log transformed) at day 192 relative to neighbour composition in the Totem Field common garden (2015-16), independent variables and those nested in other variables. Intercept represents the base value for a population that is historically exposed to herbivores with winged fruits.

| **Fixed Effects Variable** | **Estimate** | **SE** | **z-value** | **p-value** |
| --- | --- | --- | --- | --- |
| Intercept | -0.74 | 0.21 | -3.50 | < 0.001 |
| Browsing History: Historically Naïve | 0.45 | 0.06 | 7.71 | < 0.0001 |
| Fruit Phenotype of Surviving Plant: Wingless | -0.043 | 0.02 | -1.90 | 0.06 |
| Proportion of Neighbours from Historically  Exposed Populations | -0.045 | 0.06 | -0.80 | 0.43 |
| Number of Living Neighbours | 0.0043 | 0.01 | -0.32 | 0.75 |
| Mean Height of Neighbouring Plants | -0.0010 | 0.00 | 1.06 | 0.29 |
| Browsing History: Historically Naïve * Fruit  Phenotype of Surviving Plant: Wingless | -0.0091 | 0.03 | -0.32 | 0.75 |
| Proportion of Neighbours from Historically  Exposed Populations * Number of Living  Neighbours | 0.0030 | 0.01 | 0.24 | 0.81 |
| Number of Living Neighbours * Mean Height of  Neighbouring Plants | 0.00033 | 0.00 | 1.78 | 0.07 |

**Historically Exposed, plants with winged fruits:**

H:W Ratio at Day 192 = -0.74 - 0.045(proportion of neighbours from historically exposed populations) + 0.0043(# living neighbours) - 0.0010(Mean height of neighbouring plants) – 0.003(proportion of neighbours from historically exposed populations)(# living neighbours) + 0.00033 (# living neighbours)( Mean height of neighbouring plants)

**Historically Exposed, plants with wingless fruits:**

H:W Ratio at Day 192 = -0.78 - 0.045(proportion of neighbours from historically exposed populations) + 0.0043(# living neighbours) - 0.0010(Mean height of neighbouring plants) – 0.003(proportion of neighbours from historically exposed populations)(# living neighbours) + 0.00033 (# living neighbours)( Mean height of neighbouring plants)

**Historically Naïve, plants with winged fruits:**

H:W Ratio at Day 192 = -0.29 - 0.045(proportion of neighbours from historically exposed populations) + 0.0043(# living neighbours) - 0.0010(Mean height of neighbouring plants) – 0.003(proportion of neighbours from historically exposed populations)(# living neighbours) + 0.00033 (# living neighbours)( Mean height of neighbouring plants)

**Historically Naïve, plants with wingless fruits:**

H:W Ratio at Day 192 = -0.33 - 0.045(proportion of neighbours from historically exposed populations) + 0.0043(# living neighbours) - 0.0010(Mean height of neighbouring plants) – 0.003(proportion of neighbours from historically exposed populations)(# living neighbours) + 0.00033 (# living neighbours)( Mean height of neighbouring plants)


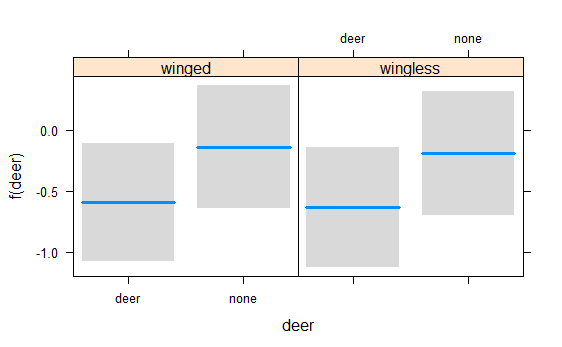


**Figure A10.1** The partial residual plot visualizing the influences of historical exposure to browsing (populations with deer or none) and fruit phenotype on the H:W Ratio at Day 192. This plot demonstrates that plants from populations without deer (none) had a lower H:W ratio than plants from populations without deer (Browsing History, p < 0.0001). There was no obvious effect of fruit phenotype in this analysis (Fruit Phenotype of Surviving Plant, p = 0.06). Plots generated using the visreg package in R.

: ANCOVA Results for Total Number of Branches Relative to Neighbour Composition in the Totem Field Common Gardens

Table A11.1 ANCOVA results for the model predicting the total number of branches relative to neighbour composition in the Totem Field common garden (2015-16), including Wald’s Chi-Square and p-values for fixed effect variables and interactions between variables, using Type III tests. Bold p-values denote statistical significance.

| **Fixed Effects Variable or Interaction** | **Df** | **χ^2^** | **p-value** |
| --- | --- | --- | --- |
| **Fixed Effects Variable** |  |  |  |
| Intercept | 1 | 152.12 | **< 0.0001** |
| Browsing History | 1 | 0.21 | 0.64 |
| Fruit Phenotype of Surviving Plant | 1 | 0.27 | 0.60 |
| Proportion of Neighbours from Historically Exposed Populations | 1 | 3.32 | 0.07 |
| Number of Living Neighbours | 1 | 84.96 | **< 0.0001** |
| Mean Height of Neighbouring Plants | 1 | 4.00 | **0.045** |
| **Two-way Interactions of Fixed Effects** |  |  |  |
| Browsing History* Fruit Phenotype of Surviving Plant | 1 | 18.49 | **< 0.0001** |
| Proportion of Neighbours from Historically Exposed Populations  * Number of Living Neighbours | 1 | 7.37 | **0.007** |
| Number of Living Neighbours * Mean Height of Neighbouring  Plants | 1 | 22.93 | **< 0.0001** |

Table A11.2 Variance accounted for by random effects in the model predicting the total number of branches relative to neighbour composition in the Totem Field common garden (2015-16).

| **Random Effects Variable** | **Variance** | **SD** |
| --- | --- | --- |
| Garden Year | 8.9 x 10^-2^ | 0.30 |
| Garden Year/Bed ID | 3.5 x 10^-2^ | 0.19 |
| Population | 0.12 | 0.35 |

Table A11.3 Estimate for each term in the model for the total number of branches relative to neighbour composition in the Totem Field common garden (2015-16), including independent variables and those nested in other variables. Estimates are with a Poisson distribution and a log link. Intercept represents the base value for a population that is historically exposed to herbivores with winged fruits.

| **Fixed Effects Variable** | **Estimate** | **SE** | **z-value** | **p-value** |
| --- | --- | --- | --- | --- |
| Intercept | 3.07 | 0.25 | 12.33 | < 0.0001 |
| Browsing History: Historically Naïve | 0.05 | 0.12 | 0.46 | 0.64 |
| Fruit Phenotype of Surviving Plant: Wingless | -0.01 | 0.03 | -0.52 | 0.60 |
| Proportion of Neighbours from Historically  Exposed Populations | 0.12 | 0.07 | 1.82 | 0.07 |
| Number of Living Neighbours | -0.15 | 0.02 | -9.22 | < 0.0001 |
| Mean Height of Neighbouring Plants | -0.002 | 0.001 | -2.00 | 0.05 |
| Browsing History: Historically Naïve * Fruit  Phenotype of Surviving Plant: Wingless | -0.14 | 0.03 | -4.30 | < 0.0001 |
| Proportion of Neighbours from Historically  Exposed Populations * Number of Living  Neighbours | 0.04 | 0.01 | 2.71 | 0.01 |
| Number of Living Neighbours * Mean Height of  Neighbouring Plants | 0.001 | 0.00 | 4.79 | < 0.0001 |

**Historically Exposed, plants with winged fruits:**

Total # Branches = 3.07 + 0.12 (proportion of neighbours from historically exposed populations) - 0.15(# living neighbours) - 0.002(Mean height of neighbouring plants) –+ 0.04(proportion of neighbours from historically exposed populations)(# living neighbours) + 0.001 (# living neighbours)( Mean height of neighbouring plants)

**Historically Exposed, plants with wingless fruits:**

Total # Branches = 3.06 + 0.12 (proportion of neighbours from historically exposed populations) - 0.15(# living neighbours) - 0.002(Mean height of neighbouring plants) –+ 0.04(proportion of neighbours from historically exposed populations)(# living neighbours) + 0.001 (# living neighbours)( Mean height of neighbouring plants)

**Historically Naïve, plants with winged fruits:**

Total # Branches = 3.12 + 0.12 (proportion of neighbours from historically exposed populations) - 0.15(# living neighbours) - 0.002(Mean height of neighbouring plants) –+ 0.04(proportion of neighbours from historically exposed populations)(# living neighbours) + 0.001 (# living neighbours)( Mean height of neighbouring plants)

**Historically Naïve, plants with wingless fruits:**

Total # Branches = 2.97 + 0.12 (proportion of neighbours from historically exposed populations) - 0.15(# living neighbours) - 0.002(Mean height of neighbouring plants) –+ 0.04(proportion of neighbours from historically exposed populations)(# living neighbours) + 0.001 (# living neighbours)( Mean height of neighbouring plants)


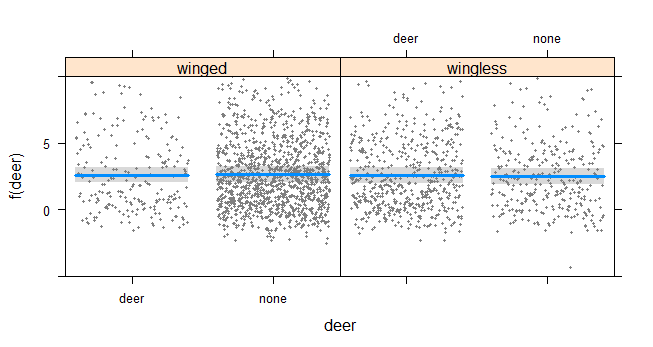


**Figure A11.1** The partial residual plot visualizing the interaction between historical exposure to browsing (populations with deer or none) and fruit phenotype for the model predicting the number of branches. This plot demonstrates that there is no obvious interaction or differences between groups, despite model significance (Browsing History* Fruit Phenotype of Surviving Plant, p < 0.0001). Plots generated using the visreg package in R.


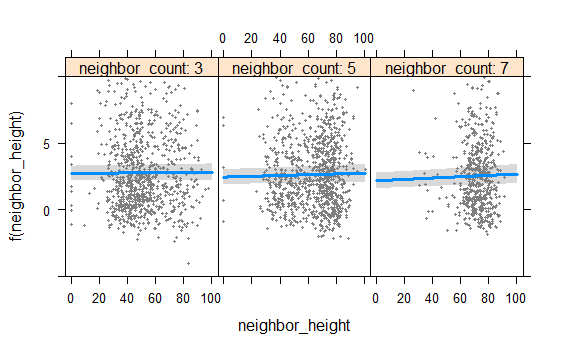


**Figure A11.2** The partial residual plot visualizing the interaction between mean height of neighbouring plants and the number of neighbouring plants for the model predicting the number of branches. This plot demonstrates that there is a significant, but minor, increase in the number of branches as the height of neighbouring plants increases, and that this pattern has a steeper slope with more neighbouring plants (Number of Living Neighbours * Mean Height of Neighbouring Plants, p < 0.0001). Plots generated using the visreg package in R.


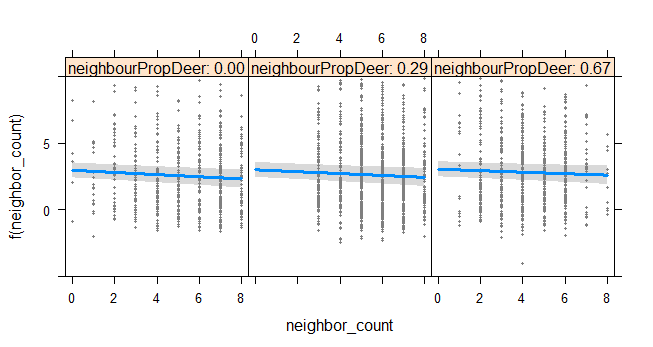


**Figure A11.3** The partial residual plot visualizing the interaction between the number of neighbouring plants and the proportion of plants from populations with deer for the model predicting the number of branches. This plot demonstrates that there is a significant, but minor, decrease in the number of branches as the number of neighbours increases (Proportion of Neighbours from Historically Exposed Populations * Number of Living Neighbours, p = 0.007). Plots generated using the visreg package in R.

: ANCOVA Results for the Number of Branches Below 10 cm Relative to Neighbour Composition in the Totem Field Common Gardens

Table A12.1 ANCOVA results for the model predicting the number of branches below 10 cm of height relative to neighbour composition in the Totem Field common garden (2015-16), including Wald’s Chi-Square and p-values for fixed effect variables and interactions between variables, using Type III tests. Bold p-values denote statistical significance.

| **Fixed Effects Variable or Interaction** | **Df** | **χ^2^** | **p-value** |
| --- | --- | --- | --- |
| **Fixed Effects Variable** |  |  |  |
| Intercept | 1 | 238.41 | **< 0.0001** |
| Browsing History | 1 | 30.53 | **< 0.0001** |
| Fruit Phenotype of Surviving Plant | 1 | 0.86 | 0.35 |
| Proportion of Neighbours from Historically Exposed Populations | 1 | 2.78 | 0.10 |
| Number of Living Neighbours | 1 | 6.51 | **0.01** |
| Mean Height of Neighbouring Plants | 1 | 0.17 | 0.68 |
| **Two-way Interactions of Fixed Effects** |  |  |  |
| Browsing History* Fruit Phenotype of Surviving Plant | 1 | 1.20 | 0.27 |
| Proportion of Neighbours from Historically Exposed Populations  * Number of Living Neighbours | 1 | 0.72 | 0.39 |
| Number of Living Neighbours * Mean Height of Neighbouring  Plants | 1 | 0.04 | 0.83 |

Table A12.2 Variance accounted for by random effects in the model predicting the number of branches below 10 cm of height relative to neighbour composition in the Totem Field common garden (2015-16).

| **Random Effects Variable** | **Variance** | **SD** |
| --- | --- | --- |
| Garden Year | 1.2 x 10^-7^ | 3.6 x 10^-4^ |
| Garden Year/Bed ID | 2.3 x 10^-2^ | 0.15 |
| Population | 6.2 x 10^-2^ | 0.25 |
| Population/Family | 0.19 | 0.44 |

Table A12.3 Estimates and modifications for each term in the model for the number of branches below 10 cm of height relative to neighbour composition in the Totem Field common garden (2015-16), including independent variables and those nested in other variables. Estimates are with a Poisson distribution and a log link. Intercept represents the base value for a population that is historically exposed to herbivores with winged fruits.

| **Fixed Effects Variable** | **Estimate** | **SE** | **z-value** | **p-value** |
| --- | --- | --- | --- | --- |
| Intercept | 2.32 | 0.15 | 15.44 | < 0.0001 |
| Browsing History: Historically Naïve | -0.57 | 0.10 | -5.53 | < 0.0001 |
| Fruit Phenotype of Surviving Plant: Wingless | -0.038 | 0.04 | -0.93 | 0.36 |
| Proportion of Neighbours from Historically Exposed  Populations | 0.17 | 0.10 | 1.67 | 0.10 |
| Number of Living Neighbours | -0.068 | 0.03 | -2.55 | 0.01 |
| Mean Height of Neighbouring Plants | -0.00075 | 0.00 | -0.41 | 0.68 |
| Browsing History: Historically Naïve * Fruit  Phenotype of Surviving Plant: Wingless | -0.063 | 0.06 | -1.10 | 0.27 |
| Proportion of Neighbours from Historically Exposed  Populations * Number of Living Neighbours | 0.019 | 0.02 | 0.85 | 0.40 |
| Number of Living Neighbours * Mean Height of  Neighbouring Plants | -0.000076 | 0.00 | -0.21 | 0.83 |

**Historically Exposed, plants with winged fruits:**

# Branches < 10 cm = 2.32 + 0.17 (proportion of neighbours from historically exposed populations) - 0.068(# living neighbours) - 0.00075(Mean height of neighbouring plants) –+ 0.0019(proportion of neighbours from historically exposed populations)(# living neighbours) - 0.000076 (# living neighbours)( Mean height of neighbouring plants)

**Historically Exposed, plants with wingless fruits:**

# Branches < 10 cm = 2.28 + 0.17 (proportion of neighbours from historically exposed populations) - 0.068(# living neighbours) - 0.00075(Mean height of neighbouring plants) –+ 0.0019(proportion of neighbours from historically exposed populations)(# living neighbours) - 0.000076 (# living neighbours)( Mean height of neighbouring plants)

**Historically Naïve, plants with winged fruits:**

# Branches < 10 cm = 1.75 + 0.17 (proportion of neighbours from historically exposed populations) - 0.068(# living neighbours) - 0.00075(Mean height of neighbouring plants) –+ 0.0019(proportion of neighbours from historically exposed populations)(# living neighbours) - 0.000076 (# living neighbours)( Mean height of neighbouring plants)

**Historically Naïve, plants with wingless fruits:**

# Branches < 10 cm = 1.65 + 0.17 (proportion of neighbours from historically exposed populations) - 0.068(# living neighbours) - 0.00075(Mean height of neighbouring plants) –+ 0.0019(proportion of neighbours from historically exposed populations)(# living neighbours) - 0.000076 (# living neighbours)( Mean height of neighbouring plants)


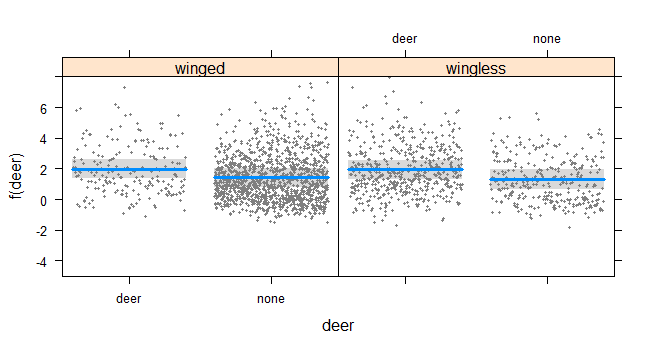


**Figure A12.1** The partial residual plot visualizing the historical exposure to browsing (populations with deer or none) and fruit phenotype for the model predicting the number of branches. This plot demonstrates that populations with deer tend to have plants with more branches below 10 cm height (Browsing History, p < 0.0001), and that there is no obvious difference by fruit phenotype (Fruit Phenotype of Surviving Plant, p =0.35). Plots generated using the visreg package in R.


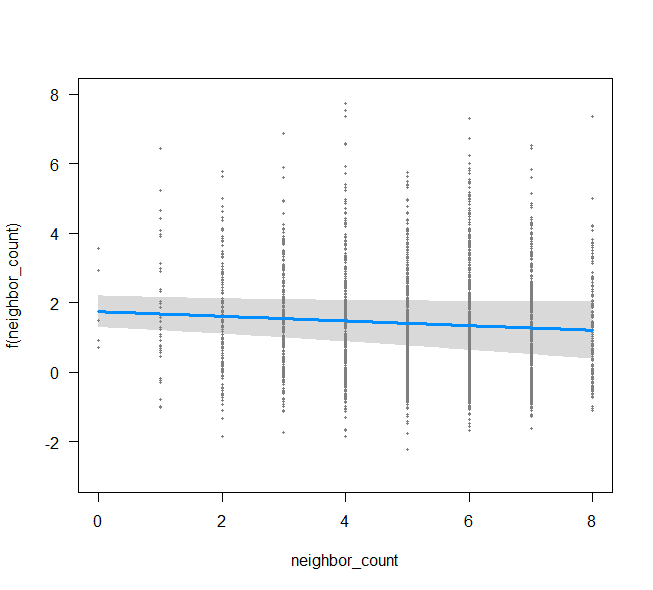


**Figure A12.2** The partial residual plot visualizing how the number of neighbours influences the number of branches below 10 cm height. This plot shows a slight, but negative trend in that as there are more neighbours, there are fewer branches below 10 cm in height (Number of Living Neighbours, p = 0.01). Plots generated using the visreg package in R.

: Calculations for the Estimated Number of Generations for Morphological Change

*N_gen_ =* [(µ_1_ - µ_2_) / µ_2_) * 100] / evolvability

*N_gen_*: number of generations; µ_1_: mean value observed for plants from historically exposed or naïve populations; µ_2_: mean value observed in the opposite selective environment

I. Plant Height Day 192:

µ_naive_ = 2.16 cm, µ_exposed_ = 1.44 cm, evolvability = 2.79-3.39

1. Number of generations from mean value historically exposed to naïve

(1.44 cm – 2.16 cm) / 2.16 cm) * 100 / 2.79 = **12 generations**

(1.44 cm – 2.16 cm) / 2.16 cm) * 100 / 3.39 = **10 generations**

1. Number of generations from mean value historically naïve to exposed

(2.16 cm – 1.44 cm) / 1.44 cm) * 100 / 2.79 = **18 generations**

(2.16 cm – 1.44 cm) / 1.44 cm) * 100 / 3.39 = **15 generations**

II. Growth Form (H:W Ratio) at Day 192:

µ_naive_ = 0.59, µ_exposed_ = 0.25, evolvability = 29.71-36.13

1. Number of generations from mean value historically exposed to naïve

(0.25 – 0.59) / 0.59) * 100 / 29.71 = **2 generations**

(0.25 – 0.59) / 0.59) * 100 / 36.13 = **2 generations**

1. Number of generations from mean value historically naïve to exposed

(0.59 – 0.25) / 0.25) * 100 / 29.71 = **5 generations**

(0.59 – 0.25) / 0.25) * 100 / 36.13 = **4 generations**

III. Number of Branches Below 10 cm Height:

µ_naive_ = 4.73, µ_exposed_ = 8.32, evolvability = 7.67 – 9.34

1. Number of generations from mean value historically exposed to naïve

(8.32 – 4.73) / 4.73) * 100 / 7.67 = **10 generations**

(8.32 – 4.73) / 4.73) * 100 / 9.34 = **8 generations**

1. Number of generations from mean value historically naïve to exposed

(4.73 – 8.32) / 8.32) * 100 / 7.67 = **6 generations**

(4.73 – 8.32) / 8.32) * 100 / 9.34 = **5 generations**
